# Therapeutic mechanisms of mulberry leaves in type 2 diabetes based on metabolomics

**DOI:** 10.3389/fphar.2022.954477

**Published:** 2022-08-30

**Authors:** Quantao Ma, Yaqi Li, Ruixue Zhao, Ziyan Tang, Jialin Li, Cong Chen, Xiaoyao Liu, Yujie Hu, Ting Wang, Baosheng Zhao

**Affiliations:** ^1^ School of Traditional Chinese Medicine, Beijing University of Chinese Medicine, Beijing, China; ^2^ School of Chinese Materia Medica, Beijing University of Chinese Medicine, Beijing, China; ^3^ Dongzhimen Hospital, Beijing University of Chinese Medicine, Beijing, China; ^4^ Beijing Research Institute of Chinese Medicine, Beijing University of Chinese Medicine, Beijing, China

**Keywords:** mulberry leaf, type 2 diabetes, metabolomics, metabolic pathways, hypoglycemic mechanism

## Abstract

**Background:** Type 2 diabetes (T2D) is considered as one of the most significant metabolic syndromes worldwide, and the long-term use of the drugs already on the market for T2D often gives rise to some side effects. The mulberry leaf (ML), *Morus alba L.*, has advantages in terms of its comprehensive therapeutic efficacy, which are characterized as multicomponent, multitarget, multipathway, and matching with the complex pathological mechanisms of diabetes.

**Methods:** T2D rats were established by a high-fat diet combined with an intraperitoneal injection of streptozotocin; an evaluation of the hypoglycemic effects of the ML in combination with fasting blood glucose and other indicators, in addition to the utilization of metabolomics technology, was performed to analysis the metabolite changes in serum of rats.

**Results:** MLs significantly reduced the fasting blood glucose of T2D rats, while improving the symptoms of polyphagia and polyuria. ML treatment altered the levels of various metabolites in the serum of T2D rats, which are involved in multiple metabolic pathways (amino acid metabolism, carbohydrate metabolism, and lipid metabolism), played a role in antioxidative stress and anti-inflammation, modulated immune and gluconeogenesis processes, and improved obesity as well as insulin resistance (IR).

**Conclusion:** The ML contains a variety of chemical components, and metabolomic results have shown that MLs regulate multiple metabolic pathways to exert hypoglycemic effects, suggesting that MLs may have great promise in the development of new hypoglycemic drugs.

## 1 Introduction

With the development of economies and the improvement of peoples’ living standards, the incidence of diabetes is increasing year by year. In 2021, the 10th edition of the Diabetes Map of the International Diabetes Federation showed that there were about 537 million diabetics in the world, and it is estimated that, by 2045, the number of diabetics may increase to 784 million (International Diabetes Federation, 2021), among which type 2 diabetes (T2D) is the majority, accounting for more than 90% of the total number of diabetic patients. T2D is a chronic metabolic disease characterized by disorders in the metabolism of glucose and lipids (abnormal blood glucose and lipid levels); the long-term use of the drugs already on the market for T2D often gives rise to some side effects (Chatterjee et al., 2017).

Traditional Chinese medicine (TCM) has a long history of treating diabetes in clinical practices in China (Gryn et al., 2013; Wei et al., 2018), which are characterized as multicomponent, multitarget, multipathway, and matching with the complex pathological mechanisms of diabetes (Chang et al., 2016; Bai et al., 2021). The mulberry leaf (ML), *Morus alba L.*, is a commonly used medicine in traditional Chinese medicine, and it has various pharmacological effects. In studies, it has been shown that the ML can resist oxidation and regulate lipid metabolism (Liu et al., 2021) as well as induce the browning of inguinal white adipose tissue through regulating the AMP-activated protein kinase signaling pathway (Cheng et al., 2022), and its therapeutic effect on diabetes has been widely recognized. Clinical studies have shown that MLs improve glycemic and insulinemic responses to sucrose in healthy subjects (Thondre et al., 2021). However, the chemical composition and mechanism of MLs in the treatment of T2D are still unclear.

The pathogenesis of T2D is complex and not yet fully understood, and it is generally believed that the pathogenesis of T2D is related to pancreatic islet cell damage, reduced insulin secretion, and insulin resistance (IR). Small-molecule metabolites and metabolic pathways are disturbed in diabetic patients, leading to abnormal body indicators and the development of diabetic complications (Turner et al., 2010; Chatterjee et al., 2017). Disorders in glucolipid metabolism further contribute to the development of T2D; exploring the changes in metabolites in ML-regulated diabetes may provide new insights into the hypoglycemic mechanisms of MLs.

The development of omics techniques, including genomics, transcriptomics, proteomics, and metabolomics, has made it possible to explore TCM’s mechanisms in the treatment of T2D (Chen and Gerszten, 2020). Metabolomics can comprehensively analyze the changes in the metabolites in body fluids, which is an effective manner of finding disease-related biomarkers and provides the possibility of exploring the mechanisms of drug treatments (Pereira et al., 2022). In recent years, some comprehensive and systematic studies have been conducted on the fluctuation in the content of metabolites from T2D patients by using metabolomics technology, looking for biomarkers and possible metabolic pathways of the pathogenesis of T2D, which provided a theoretical basis for the pathogenesis of T2D (Wang et al., 2018; Chen and Gerszten, 2020).

In order to fully understand the chemical composition and hypoglycemic mechanisms of MLs, UPLC-Q-Exactive Orbitrap-MS was used to explore the chemical composition of MLs, and a metabolomics technique was used to explore the abundance of changes in the metabolites in the serum of rats after treatment with MLs. This study provides a new idea for the prevention and treatment of T2D in addition to a new vision for the development of new hypoglycemic herbal medicines based on MLs.

## 2 Materials and methods

### 2.1 Chemicals and reagents

Mulberry leaf (no. 20180120, Beijing Tongrentang Co., Ltd., China), metformin (no. AAT8173, Sino-US Shanghai Squibb Pharmaceuticals Co., Ltd., China), streptozotocin (no. WXBB6772V, Sigma, United States), citrate buffer solution, sodium pentobarbital (no. 2018020, Merck, Germany), 10% neutral formalin fixation solution (no. 20181115, Tianjin Yili Chemical Reagent Co., Ltd., China), methanol, acetonitrile, formic acid (ThermoFisher Scientific, Inc., United States), ammonium acetate, and ammonia (Shanghai Aladdin Biochemical Technology Co., Ltd., China).

Isoquercitrin (no. 111809-201804), 1-deoxynojirimycin (no. 112008-201801), caffeic acid (no. 110885-201703), 7-hydroxycoumarin (no. 111739-200501), β-sitosterol (no. 110851-201909), threonine (no. 140682-201302), phenylalanine (no. 140676-201706), vitamin B1 (no. 100390-201806), resveratrol (no. 111535-201703), and scopolamine lactone (no. 110768-200504); all of the above were purchased from the China Academy of Food and Drug Inspection and Quarantine.

A high-sugar and high-fat feed (feed formula: 20% sucrose, 15% lard, 1.2% cholesterol, 0.2% sodium cholic acid, and an appropriate amount of casein, calcium hydrogen phosphate, and stone powder) was purchased from Beijing Huafucang Biotechnology Co., Ltd.

### 2.2 Equipment

A centrifuge (ThermoFisher Scientific, United States), blood glucose meter, blood glucose test paper (Shanghai Qiangsheng Medical Equipment Co., Ltd., China), rotary evaporator (BUCHI, Switzerland), constant-temperature water bath pot (Shanghai Yiheng Scientific Instrument Co., Ltd., China), ultrahigh-performance liquid chromatograph (DionexTM UltimateTM 3000, ThermoFisher Scientific, United States), quadrupole orbital ion mass spectrometer (Q ExactiveTM, ThermoFisher Scientific, United States), desktop high-speed cryocentrifuge (ThermoFisher Scientific, United States), freeze dryer (CHRIST company, Germany), scroll oscillator (SI instrument company, United States), and a reversed-phase T3 chromatographic column (ACQUITY UPLC BEH, T3, 2.1 × 100 mm, 1.7 μm, Waters, United States).

### 2.3 Preparation of MLs

Preparation of ML powder: the MLs (2.0 kg) were soaked in deionized water (20 L) for 30 min and extracted at 85–95°C for 2.5 h. The concentrated extracts were placed into a vacuum oven and dried into powder. The powder was weighed and the yield was calculated (yield: 29.55%).

Sample pretreatment of MLs of LC-MS: 0.5 g of the ML powder was weighed and placed in a volumetric flask, 50 ml of methanol was added, it was sonicated for 30 min, it was cooled down and the lost weight was made up for with methanol, it was filtered with a 0.22 μm microporous filter membrane, and then the continued filtrate was taken.

Sample pretreatment of standard compounds for LC-MS:10 mg of each compound was weighed in a 10 ml volumetric flask, dissolved with methanol, and shaken well to prepare a solution with a concentration of 1 mg/ml. Of each compound solution, 1 ml was taken into a 10 ml volumetric flask, mixed into a mixed standard solution containing 10 compounds, and filtered through a 0.22 μm microporous membrane to obtain the filtrate.

### 2.4 Quantitative analysis of MLs

The chemical composition of MLs was determined according to the optimized liquid chromatography and mass spectrometry method. A UPLC column (ACQUITY UPLC HSS, T3, 2.1 × 100 mm, 1.8 μm, Waters), with a column temperature of 45°C, a flow rate of 0.3 ml/min, and an injection volume of 3 μL was used; the mobile phase of solution A (acetonitrile), solution B (0.1% formic acid in H_2_O), and the gradient elution, as well as the gradient elution conditions, were as follows: 0–3 min (5% A), 3–45 min (5–85% A), 45–45.1 min (85–5% A), 45.1–50 min (5% A).

The mass spectrometry was performed with a heated electrospray ion source (HESI), positive and negative ion detection modes, a Fourier transform high-resolution full scan (TF, full scan, resolution 70000), a data-dependent ddMS^2^ (data-dependent acquisition), and a parent ion list, PIL-MS2. Various strategies, such as CID fragmentation, were applied, and the scan range was 120.00–1800.00 Da. The mass spectrometry conditions of positive ion detection modes were as follows: The spray and capillary voltages were set to 4.0 KV and 35.0 V, respectively. The tube lens voltage was 55 V, and the ion source temperature was set to 400°C. Nitrogen (purity >99.99%) was used as both the sheath gas (35 arb) and the auxiliary gas (10 arb) The mass spectrometry conditions of negative ion detection modes were as follows: The spray and capillary voltages set to 3.0 kV and 35.0 V, respectively. The tube lens was set to 55 V, and the ion source temperature was set to 300°C. Nitrogen (purity >99.99%) was used as both the sheath gas (35 arb) and the auxiliary gas (10 arb). The sample assay profiles were compared with the standard profiles, compound database, and the contents of reports in the literature to infer the structures of the measured compounds. They were also combined with a multivariate statistical analysis to determine the chemical composition of MLs.

### 2.5 Establishment and administration of T2D rats

Healthy male SD rats (body weight of 200–220 g) were purchased from SPF (Beijing) Biotechnology Co., Ltd. and raised in a specific pathogen-free animal laboratory of the Beijing University of Chinese Medicine (temperature of 20–24°C, relative humidity of 50%–70%, and light/dark rhythm of 12 h/12 h). After adaptive feeding for 1 week, rats were randomly divided into control and T2D groups. The control rats were fed with a regular diet, and the T2D rats were fed with a high-sugar and -fat diet. After 4 weeks, the T2D rats had fasted for 12 h, and a 1% STZ citrate buffer solution (0.1 mmol/L, pH = 4.2–4.5, 4°C) was injected intraperitoneally with a dose of 35 mg/kg. The control rats were intraperitoneally injected with the same amount of the citrate buffer solution. On the seventh and eighth day after injection, the FBG was measured by tail vein blood sampling. The T2D rats were established successfully when the FBG ≥12 mmol/L; the unqualified rats were excluded. The T2D rats were randomly divided into a T2D group, a metformin (0.2 g/kg) group, and an ML treatment (4.0 g/kg) group according to their FBG and body weight (10 rats in each group). The rats in each group were given an intragastric administration once a day for 12 consecutive weeks; the rats in the control and T2D groups were given the same amount of distilled water.

### 2.6 Metabolomics methods

#### 2.6.1 Sample collection

After 12 weeks of administration, the rats were anesthetized by an intraperitoneal injection of 1% pentobarbital sodium (40 mg/kg). Blood was collected from the abdominal aorta, placed at room temperature for 2 h, and centrifuged at 3500 rpm for 10 min; the upper serum was taken and stored at −80°C.

#### 2.6.2 Sample pretreatment

Of the serum, 200 μL was put into a 1.5 ml centrifuge tube, and 800 μL of a chromatographic-grade precooled methanol/acetonitrile solution (v:v = 1:1) was added, which was mixed by a rapid vortex for 30 s, stood at 4°C for 30 min, and centrifuged at 12000 rpm at 4°C for 20 min. The supernatant, with a volume of 600 μL, was transferred to a new 1.5 ml centrifuge tube and freeze-dried. The freeze-dried sample was redissolved in 300 μL of a precooled acetonitrile aqueous solution (v:v = 1:1), and then was fully vortexed–oscillated. Samples were centrifuged at 12000 rpm at 4°C for 10 min, and 200 μL of the supernatant was transferred to a liquid bottle for a nontargeted LC-MS metabolomics analysis. Each sample, with a volume of 20 μL, was mixed into a 1.5 ml liquid vial to form a quality control (QC) sample.

#### 2.6.3 UPLC-Q-Exactive Orbitrap-MS analysis

The samples were separated by ThermoFisher Scientific U3000 rapid liquid chromatography. Serum samples were detected with a reversed-phase T3 chromatographic column, the metabolites in samples with a weak polarity were eluted, and as much of the metabolite information in the samples as possible was collected.

Samples were analyzed by a Waters ACQITY UPLC BEN, T3, 2.1 × 100 mm, 1.7 μm column. Mobile phase A is water containing 0.1% formic acid, and mobile phase B is pure acetonitrile. The sample injection volume was 3 μL, the flow rate was controlled as 0.3 ml/min, and the mass spectrum scanning mode was as follows: positive and negative ion scanning, the range was 80–1200 m/z, the capillary voltage and tube lens voltage were −110 V, the capillary temperature was 350°C, and the multistage mass spectrum collision normalized energy was 40 V, 20 V, and 10 V, respectively. Data dependency scan mode. The procedures of the liquid mass gradient in positive and negative ion modes were as follows: 0–1 min (95% A), 1–9 min (95–60% A), 9–19 min (60–10% A), 19–19.1 min (10–95% A), and 19.1–24 min (95% A). During the whole analysis process, samples were placed in an automatic sampler at 4°C. The QC samples were continuously injected on five occasions to balance the liquid mass system and then tested in sequence. For every five samples tested, a QC test was performed, and the stability of the system was evaluated based on the results of the QC samples.

The mass spectrometer adopted a quadrupole orbital ion trap mass spectrometer with a thermoelectric spray ion source. The ion source voltage was 3.7 kV, the capillary heating temperature was 320°C, the volumetric heating and evaporation temperatures were 300°C, the sheath gas and auxiliary gas were nitrogen, the sheath gas pressure was 30 psi, the auxiliary gas pressure was 10 psi, and the collision gas was nitrogen. The pressure was 1.5 mTorr, and the first-stage full scanning parameters were the following: the resolution was 70000, the automatic gain control target was 1×10^6^, the cycle time was 50 ms, and the scanning range was 80–1200 m/z.

#### 2.6.4 Data extraction and multivariate analysis

The original data were imported into Progenesis QI (Waters) software for data preprocessing, peak alignment, deconvolution, peak extraction, multivariate statistical analysis, and identification. After the raw data were imported, the automatic mode was selected for data processing, the mass score correction was performed through Lockmass, and the QC samples were used as the reference group for peak alignment.

All of the metabolites were identified by a comprehensive scoring of their “score”, “fragmentation score”, “mass error (PPM)”, and “isotope similarity” according to databases. The databases were HMDB (http://www.HMDB.ca), KEGG (http://www.genome.jp/), and NIST (https://chemdata.nist.gov), and the quality errors used in searching the databases were 0.005 Da (ms1) and 15 ppm (ms2) (Huang et al., 2013; Want et al., 2013; Wishart et al., 2018). The processed data were imported into the SIMCA 14.1 software, and principal component analysis (PCA) was used to cluster the data of each group; orthogonal partial least squares discriminant analysis (OPLS-DA) was selected to filter signals unrelated to classification, namely orthogonal signals, to obtain the OPLS-DA model, and VIP score screening can be performed for metabolites through model analyses. The higher the VIP score of metabolites, the more significant their contribution to the grouping. *p*-values were calculated by a Student’s t-test with SPSS 22.0 software. The metabolites with a coefficient of variation (CV) less than 30%, a fold change >1.2, a VIP >1, and a *p*-value < 0.05 were selected as differential metabolites (the levels of differential metabolites were significantly different in the serum of rats of two groups), and SIMCA 14.1 software was used to make a volcano map of differential metabolites. Metabolic pathways were analyzed by using the Metabolites Biological Role (http://csbg.cnb.csic.es/mbrole2/) website.

### 2.7 Statistical analysis

Experimental data were expressed as mean ± standard deviation (SD), and the statistical analysis of the data was performed using SPSS 22.0 software. *p*-values of <0.05 (*), <0.01 (**) were adopted for statistical significance; fasting blood glucose, body weight, water intake, and food intake data were analyzed using one-way ANOVA.

## 3 Results

### 3.1 Chemical composition identification of MLs

**FIGURE 1:**
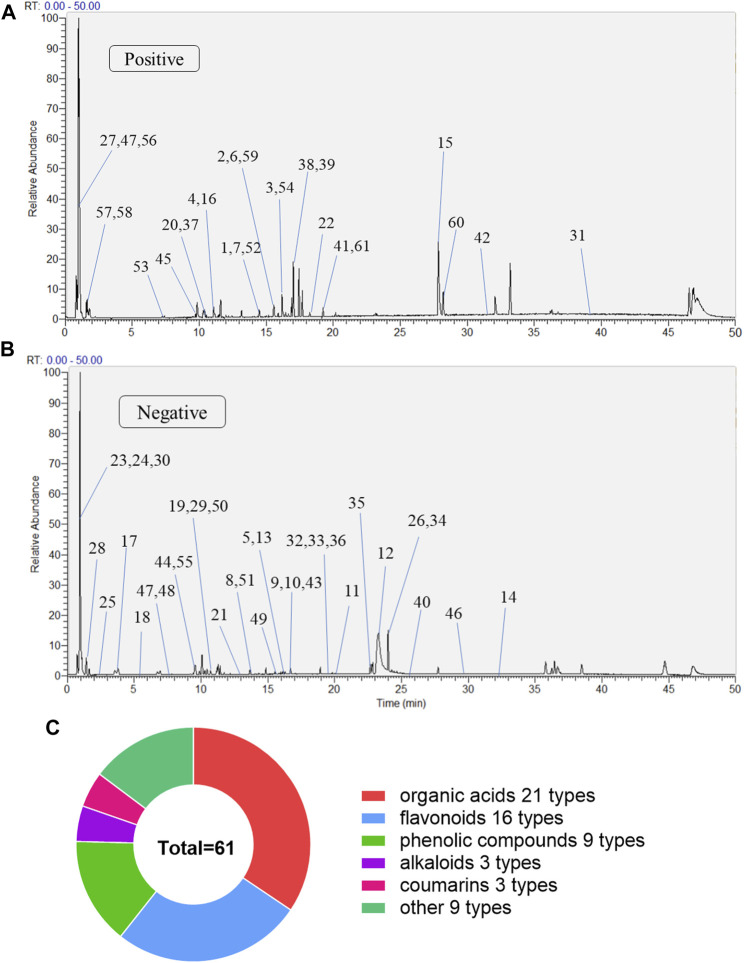
The compositions of MLs were determined by UPLC-Q-Exactive Orbitrap-MS **(A)** Total ion flow diagram of MLs in a positive mode **(B)** Total ion flow diagram of MLs in a negative mode **(C)** Number of monomer components in MLs identified by positive and anion UPLC-Q-Exactive Orbitrap-MS.

The MLs were detected using UPLC-Q-Exactive Orbitrap-MS, and the total ion chromatography (TIC) in positive and negative ion modes was obtained as shown in Figure 1. Combined with the compound structure, databases, and the literature, a total of 61 compounds were identified in the ML extracts, including 27 in a positive ion mode and 34 in a negative ion mode. They contained 16 flavonoids, 21 organic acids, nine phenolic compounds, three alkaloids, three coumarins, and nine other categories; the qualitative tables for the 61 compounds are shown in Supplementary Table S1.

#### 3.1.1 Flavonoids

A total of 16 flavonoids were identified in MLs, including rutin (Ceymann et al., 2011) (1), quercetin (Lee et al., 2012) (2), kaempferol (Li Q et al., 2022), luteolin (4), cymaroside (Yang et al., 2019) (5), kaempferol-3-o-rutinoside (Vieira et al., 2017) (6), hyperoside (7), isoquercetin (8), quercitrin (9), astragalin (10), morin (11), moracin O (12), moracin M (13), kuwanon C (14), isobavachalcone (15), and sakuranetin (16).

**FIGURE 2:**
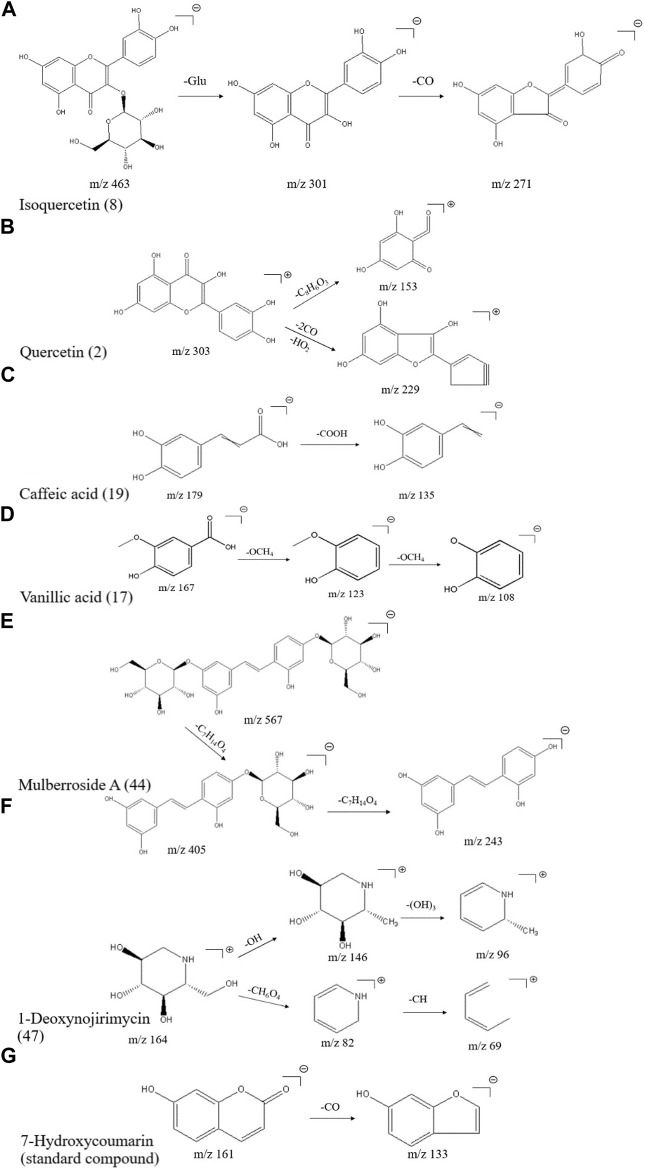
Mass spectrometric cleavage patterns of some chemical components of MLs **(A)** Isoquercitrin **(B)** quercetin **(C)** caffeic acid **(D)** vanillic acid **(E)** mulberroside A **(F)** 1-deoxynojirimycin, and **(G)** 7-hydroxycoumarin.

The elemental composition of isoquercitrin (standard compound) is C_21_H_20_O_12_ (ppm = −1.73), which shows its molecular ion peak in a HESI negative ion mode at m/z 463.08899, and produces m/z 301.03 plasma fragments in MS2, suggesting that isoquercitrin breaks the glycosidic bond at the C-ring 3-position hydroxyl group, generating m/z 301 [M-H-C_6_H_11_O_5_]^-^ fragments, followed by m/z 271 [M-H-C_6_H_11_O_5_-CO]^-^ fragments, and the cleavage behavior is shown in Figure 2A. Combined with the standard compound quality spectrum cleavage pattern, it is presumed that compound 8 is isoquercitrin.

The molecular ion peak [M + H]^+^ of compound 2 is m/z 303.05032, presumably with an elemental composition of C_15_H_10_O_7_ (ppm = -1.32). The secondary mass spectrum [M-H]^-^ yielded fragment ions m/z 229.05 [M + H-2CO-HO_2_]^+^ and m/z 153.02 [M + H-2C8H6O_3_]^+^; together with the literature (Yi et al., 2017), compound 2 is presumed to be quercetin, and the cleavage pattern is shown in Figure 2B.

#### 3.1.2 Organic acid compounds

A total of 21 organic acid compounds were identified, including vanillic acid (Klejdus and Kováčik, 2016) (17), protocatechuic acid (Yang, 2017) (18), caffeic acid (19), chlorogenic acid (Klejdus and Kováčik, 2016) (20), 2-hydroxycinnamic-acid (Yang, 2017) (21), trans-cinnamic acid (22), quinic acid (23), 2,3,4-trihydroxybenzoate (24), 3,4,5-trihydroxybenzoic acid (25), myristic acid (26), 4-guanidinobutyric acid (27), malic acid (28), shikimic acid (29), citric acid (30), α-linolenic acid (31), isochlorogenic acid C (32), isochlorogenic acid B (33), 9-12-dioxododecanoicacid (34), corchorifatty acid F (35), abscisic acid (36), and cryptochlorogenic acid (37).

The elemental composition of caffeic acid (standard compound) is C_9_H_8_O_4_ (ppm = 0.06), and its molecular ion peak in a HESI negative ion mode shows m/z 179.03497, which produces m/z 135.05 fragments in MS2, suggesting that it drops a carboxyl group to produce 135.03 [M-H-COOH]^-^ fragments, and the specific cleavage process is shown in Figure 2C. The cleavage pattern of compound 19 is presumed to be caffeic acid.

The molecular ion peak [M-H]^-^ of compound 17 was m/z 167.03462, presumably of elemental composition C_8_H_8_O_4_ (ppm = 2.16), which yielded m/z 123.04523 [M-H-OCH_4_]^-^ as well as m/z 108.02171 [M-H-OCH_4_]^-^ fragment ions in MS/MS; combined with the literature, compound 17 was presumed to be isovanillic acid, and the cleavage pattern is shown in Figure 2D.

#### 3.1.3 Phenolic compounds

A total of nine phenolic compounds were identified, including eugenol (38), syringaresinol (Morimoto et al., 1986) (39), moracin A (40), moracin P (41), moracin C (42), 4-[2-(3,5-dihydroxyphenyl)ethenyl]benzene-1,3-diol (43), mulberroside A (44), oxyresveratrol 2-O-β-d-glucopyranoside (45), and 6-gingerol (46). The molecular ion peak [M-H]- of compound 44 was m/z 567.17242, and the secondary mass spectrum showed a high abundance of m/z 405.12036 [M-H-162]^-^, 243.06654, and other fragment ions, which may be oxidized in a similar manner as resveratrol; combined with chemical professional databases and the related literature (Hirakura et al., 2004; Ai et al., 2017), it is presumed that the compound is mulberry bark glucoside A. The cleavage pattern is shown in Figure 2E.

#### 3.1.4 Alkaloids

A total of three alkaloids were identified, including 1-deoxynojirimycin (47), trigonelline (48), and liriodendrin (49). The elemental composition of 1-deoxynojirimycin (standard compound) is C_6_H_13_NO_4_ (ppm = −0.67), which shows its molecular ion peak in a HESI positive ion mode as m/z 164.09184, producing fragments of m/z 146.08, 82.07, and 69.03 in MS2, suggesting that 1-deoxynojirimycin first drops a hydroxyl group to generate a 146.08 [M + H-OH]^+^ fragment, followed by a 96.05 [M + H-(OH)_3_]^+^ fragment; meanwhile, 1-deoxynojirimycin may drop a CH6O4 to generate a 82.07 [M + H-CH_6_O_4_]^+^ fragment, followed by a 69.03 [M + H-CH_6_O_4_-CH]^+^ fragment (Xiong et al., 2021). The cleavage behavior is shown in Figure 2F.

#### 3.1.5 Coumarins

A total of three coumarins were identified, including 5,7-dihydroxycoumarin (50), skimmetin (51), and scopoletin (52). The 7-hydroxycoumarin (standard compound), as a simple coumarin molecule with an elemental composition of C_9_H_6_O_3_ (ppm = -0.12), showed its molecular ion peak in a HESI negative ion mode at m/z 161.02443, producing almost no fragmentation in MS2, and a small peak in the second mass spectrum with the molecular ion peak as the base peak, producing a small peak of 133.03 [M-H-CO]^-^ fragmentation; the cleavage pattern is shown in Figure 2G.

#### 3.1.6 Other classes of compounds

A total of nine other classes of compounds were also identified, including cinnamyl alcohol (Yang, 2021) (53), cinnamaldehyde (54), vanillin (55), l (+)-rhamnose monohydrate (56), linamarin (57), guanosine (58), ligustilide (59), phytosphingosine (60), and psychosine (61).

### 3.2 Hypoglycemic efficacy of the mulberry leaf on T2D rats

**FIGURE 3:**
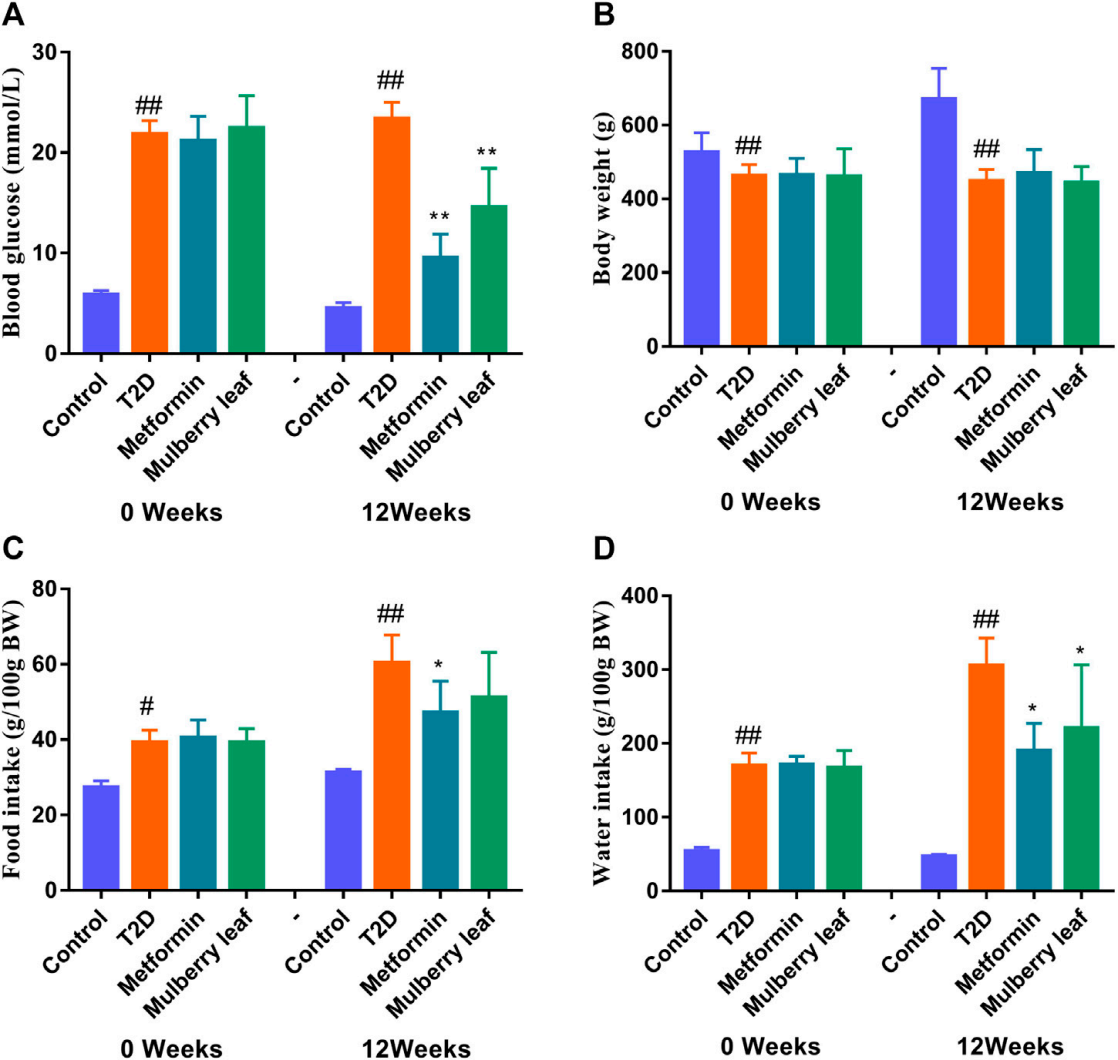
Fasting blood glucose, body weight, and food as well as water intake of rats before and after the ML treatment **(A)** Fasting blood glucose **(B)** fasting body weight **(C)** food intake, and **(D)** water intake. Data were presented as the mean ± SEM, n ≥ 7. ^#^ *p* < 0.05 vs. the control group, ^##^ *p* < 0.01 vs. the control group, ^*^ *p* < 0.05 vs. the T2D group, and ^**^ *p* < 0.05 vs. the T2D group.

Our previous study showed that MLs significantly improve the oral glucose tolerance test (OGTT), the insulin tolerance test (ITT), serum insulin, and other indexes in T2D rats, regulate blood glucose metabolism, improve glucose tolerance, enhance pancreatic islet function, improve insulin resistance, reduce blood lipid level, inhibit the expression of inflammatory factors, and improve T2D symptoms (Tian et al., 2017; Tian et al., 2019; Wang et al., 2019; Cheng et al., 2022). Therefore, the fasting blood glucose (FBG), body weight, and food as well as water intake of rats were measured in this experiment. As shown in Figure 3, MLs significantly reduced the FBG of T2D rats and improved the symptoms of excessive water intake in T2D rats. Additionally, there was a trend towards a reduction in the food intake of rats in the ML group, but no statistical difference was observed. The statistical results are presented in the Supplementary Table S2-S5.

### 3.3 Serum metabolomics profile analysis of the ML treatment of T2D

**FIGURE 4:**
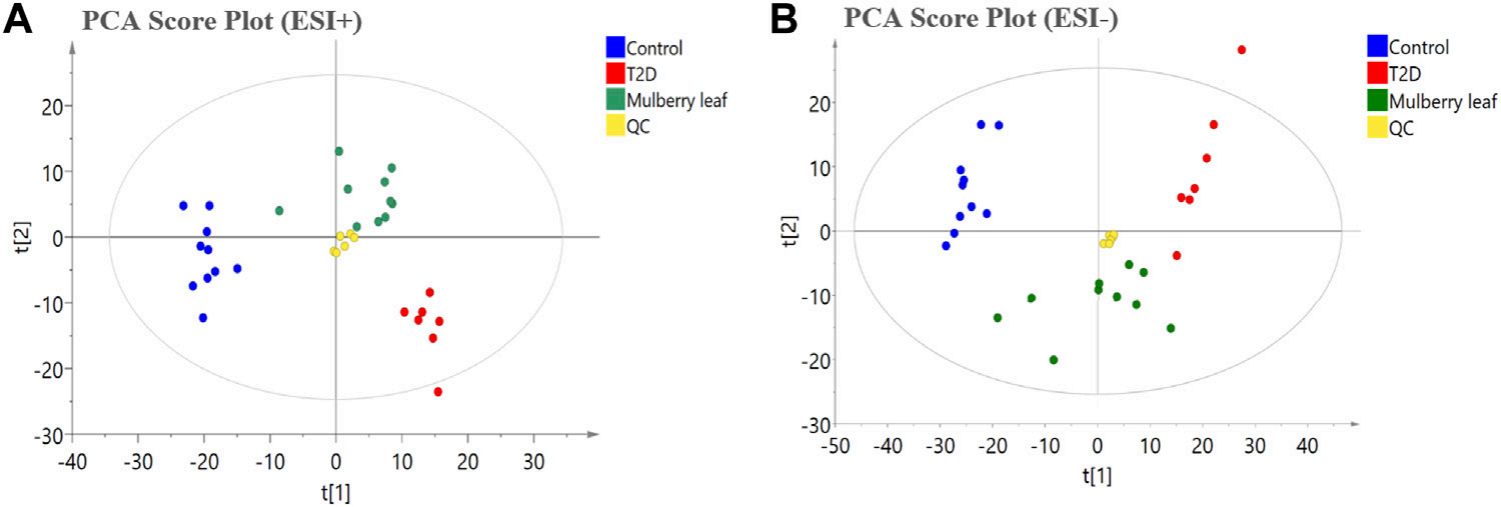
The principal component analysis (PCA) score plot of the serum metabolomics analysis **(A)** ESI + model **(B)** ESI- model.

In this study, PCA was used to observe the overall change trend in the data of each group. The PCA results showed that all of the samples were distributed within a 95% confidence interval, indicating that the PCA model was reliable. The PCA score showed that the samples of the control and T2D groups could be separated significantly under positive and negative ions, suggesting that the metabolite level in the serum of T2D rats had significant changes and that the physiological metabolism in the serum was disturbed. The metabolites in the serum of the rats in the ML group were significantly different from those in the T2D group, and tended to recover to those of the control group, suggesting that MLs regulate the metabolic process and improve the metabolic disorder in T2D rats (Figure 4).

**FIGURE 5:**
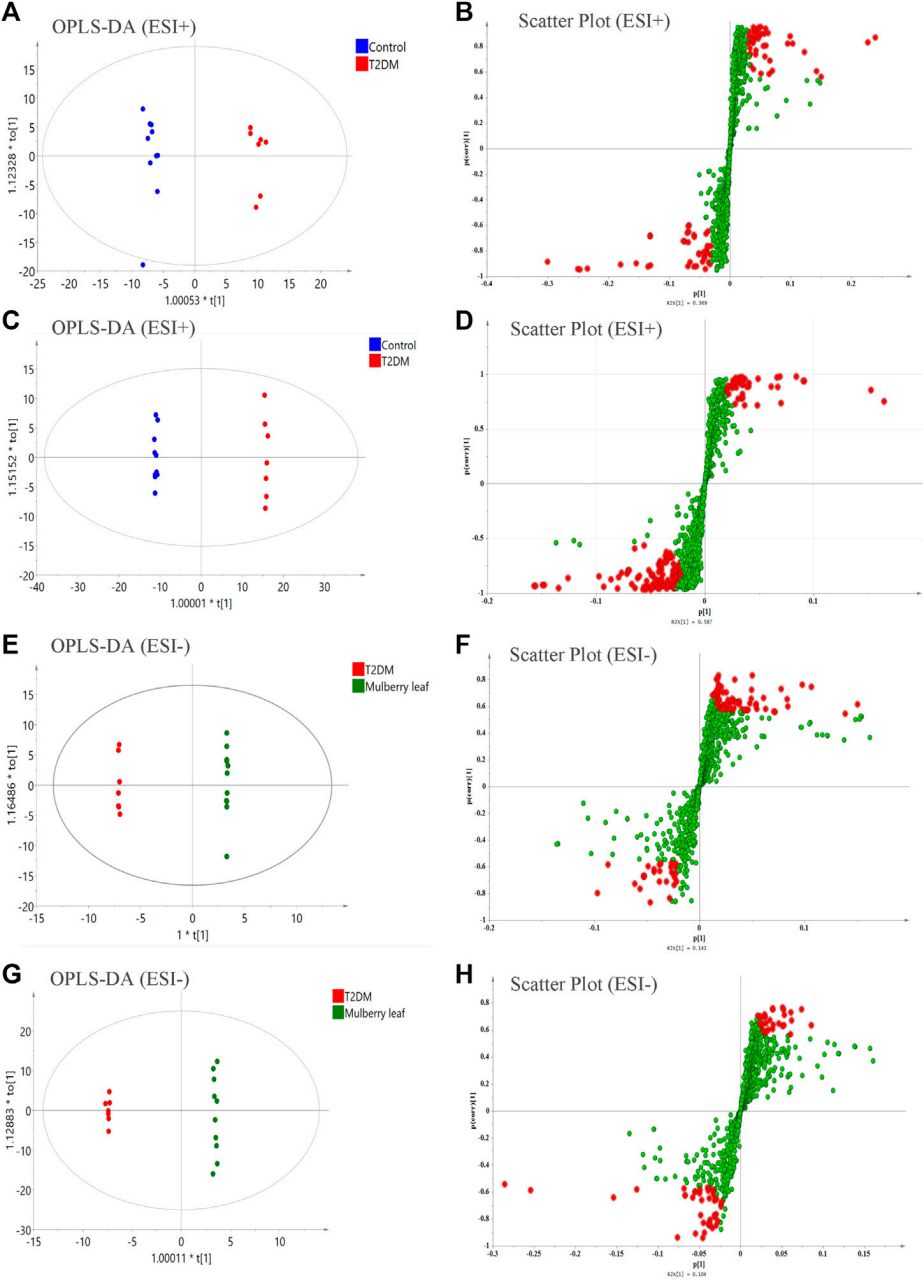
The orthogonal partial least squares discriminant analysis (OPLS-DA) score plots, S-plots of the serum metabolomics analysis **(A–D)** ESI + model **(E–H)** ESI- model.

Then, a supervised OPLS-DA model was used to compare each group in pairs (Figure 5). All of the identified differential metabolites were summarized to determine the differences in the metabolites in the serum of rats between the T2D group and the control group as well as the ML group and the T2D group (VIP >1, fold change >1.2, and *p*-value < 0.05), and look for metabolites whose metabolic levels are reversed by MLs. Finally, 88 endogenous differential metabolites were obtained in the serum, of which 62 were upregulated and 26 downregulated (Table S6).

### 3.4 Pathway enrichment and mechanisms analysis

**FIGURE 6:**
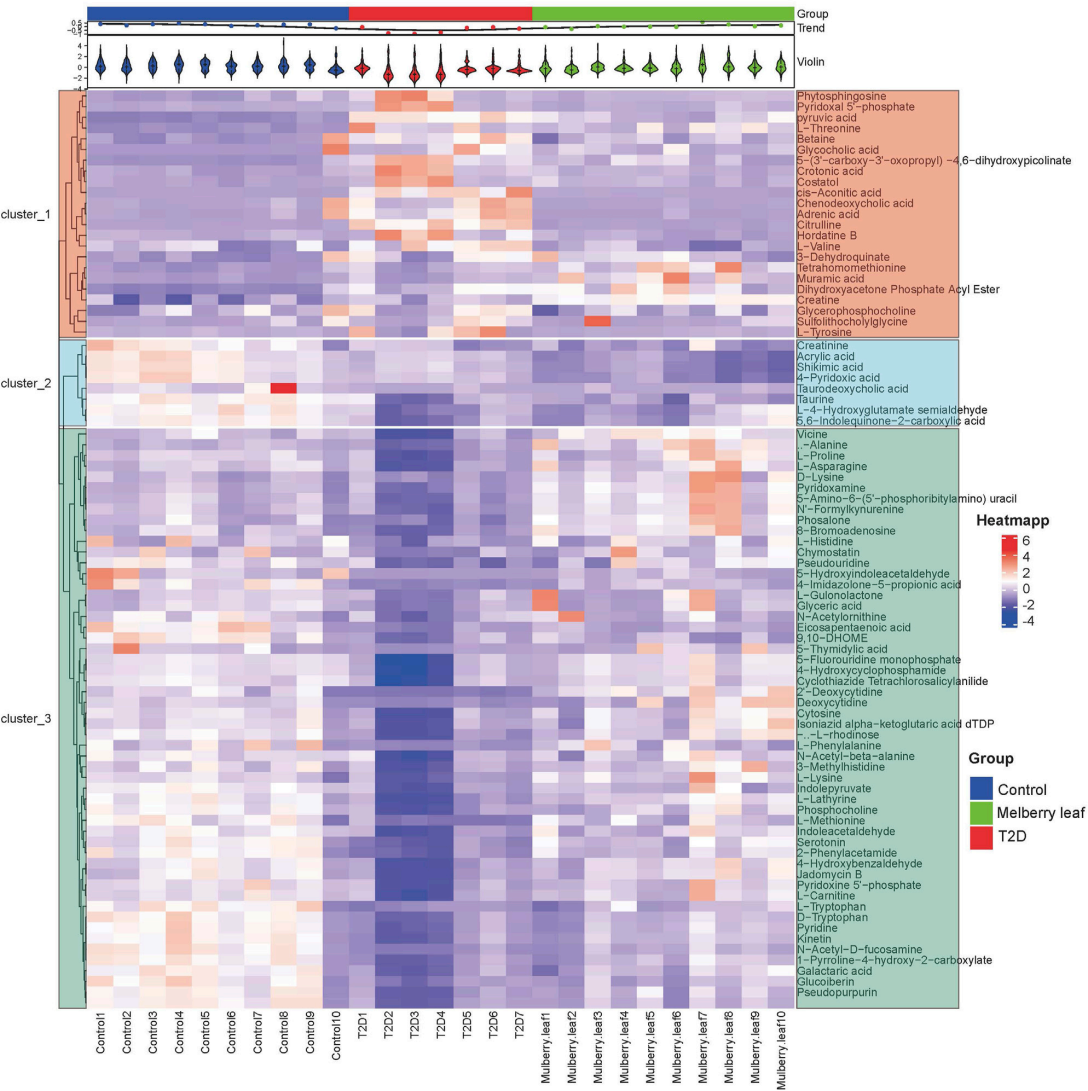
Heat map of the differential metabolites in the serum of rats (Red and blue represent increased and decreased metabolite content, respectively).

A heat map analysis of the potential differential metabolites in the serum of rats was conducted. The results showed that the levels of metabolites in the serum were significantly different between the control and T2D rats, and that the levels in the ML group tended to recover to those of the control group, suggesting that MLs could correct the abnormal levels of serum metabolites in T2D rats, as shown in Figure 6.

**FIGURE 7:**
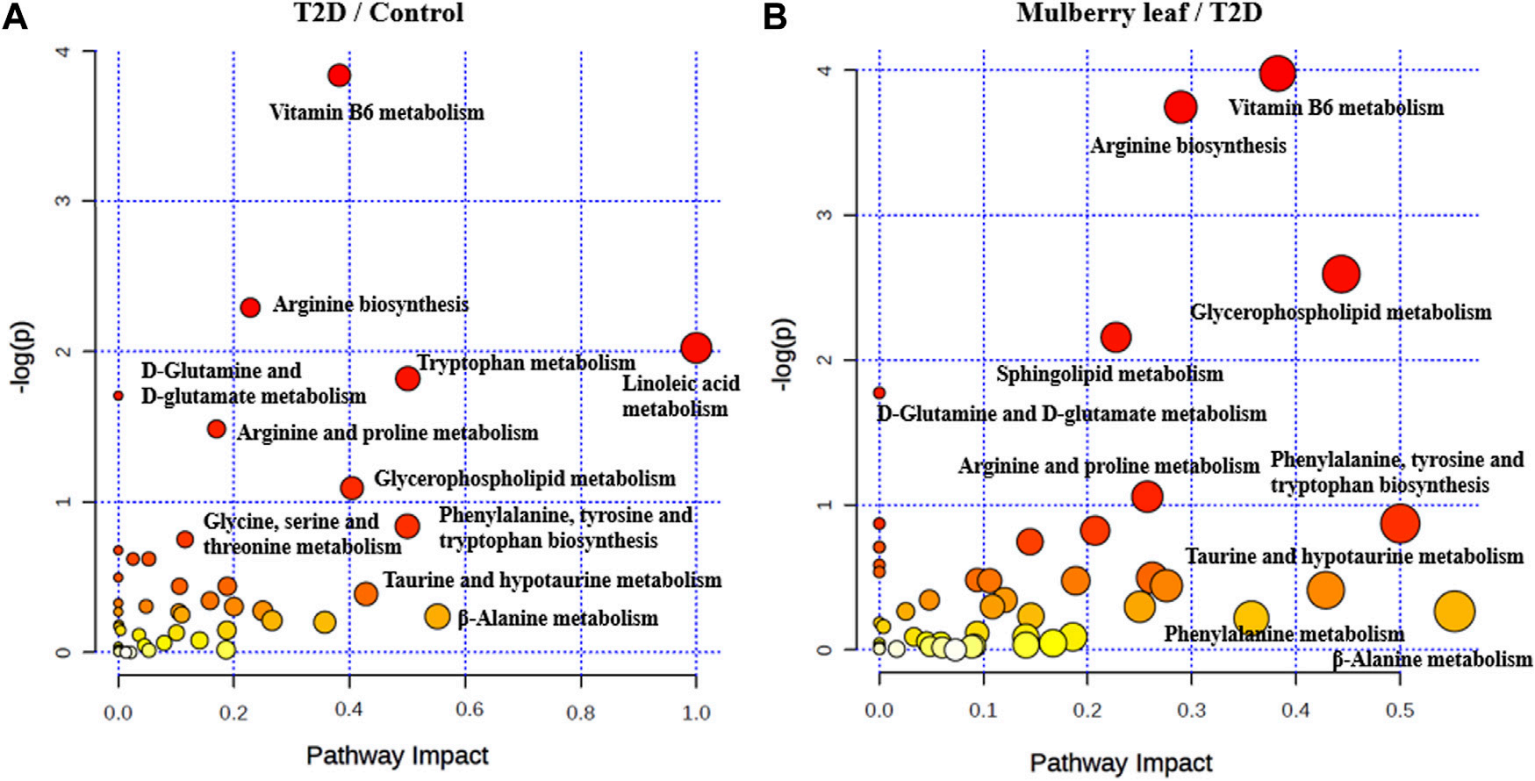
Bubble diagram of the differential metabolite pathway analysis in the serum of rats **(A)** T2D vs. control **(B)** ML vs. T2D.

Pathway enrichment was carried out for the metabolites with significant changes in the serum of rats, as shown in Figure 7. In the picture, each circle represents a different metabolic pathway, and the color of the circle represents the -log (*p* value), which is also represented along the vertical axis. The horizontal axis represents the enrichment factor; the greater the enrichment factor, the higher the enrichment level of the differential metabolites in this pathway. The pathway on the upper right of the figure is the significantly affected differential pathway.

**FIGURE 8:**
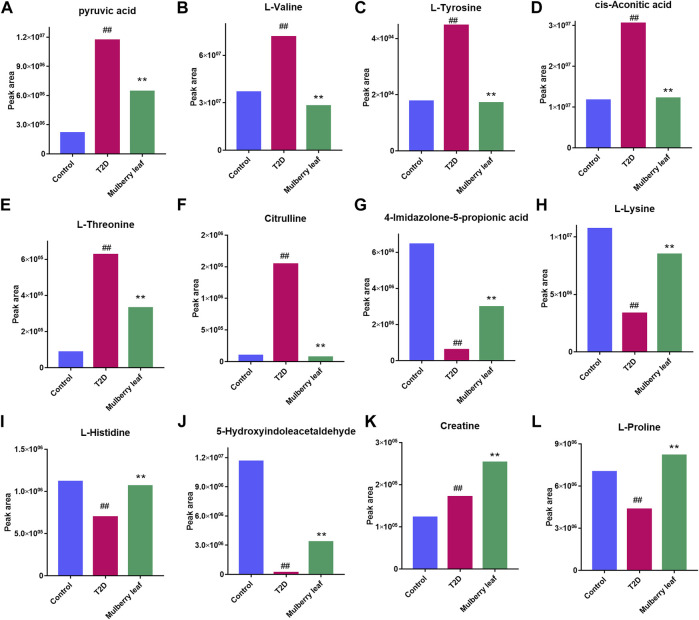
Potential metabolite changes in the serum of T2D rats with the ML treatment **(A)** Pyruvic acid **(B)** l-valine **(C)** l-tyrosine **(D)** Cis-aconitic acid **(E)** l-threonine **(F)** Citrulline **(G)** 4-Imidazolone-5-propionic acid **(H)** l-lysine **(I)** l-histidine **(J)** 5-Hydroxyindoleacetaldehyde **(K)** Creatine **(L)** l-proline (^##^ *p* < 0.01 vs. the control group. ^**^ *p* < 0.01 vs. the T2D group. ML: (4 g/kg).).

**FIGURE 9:**
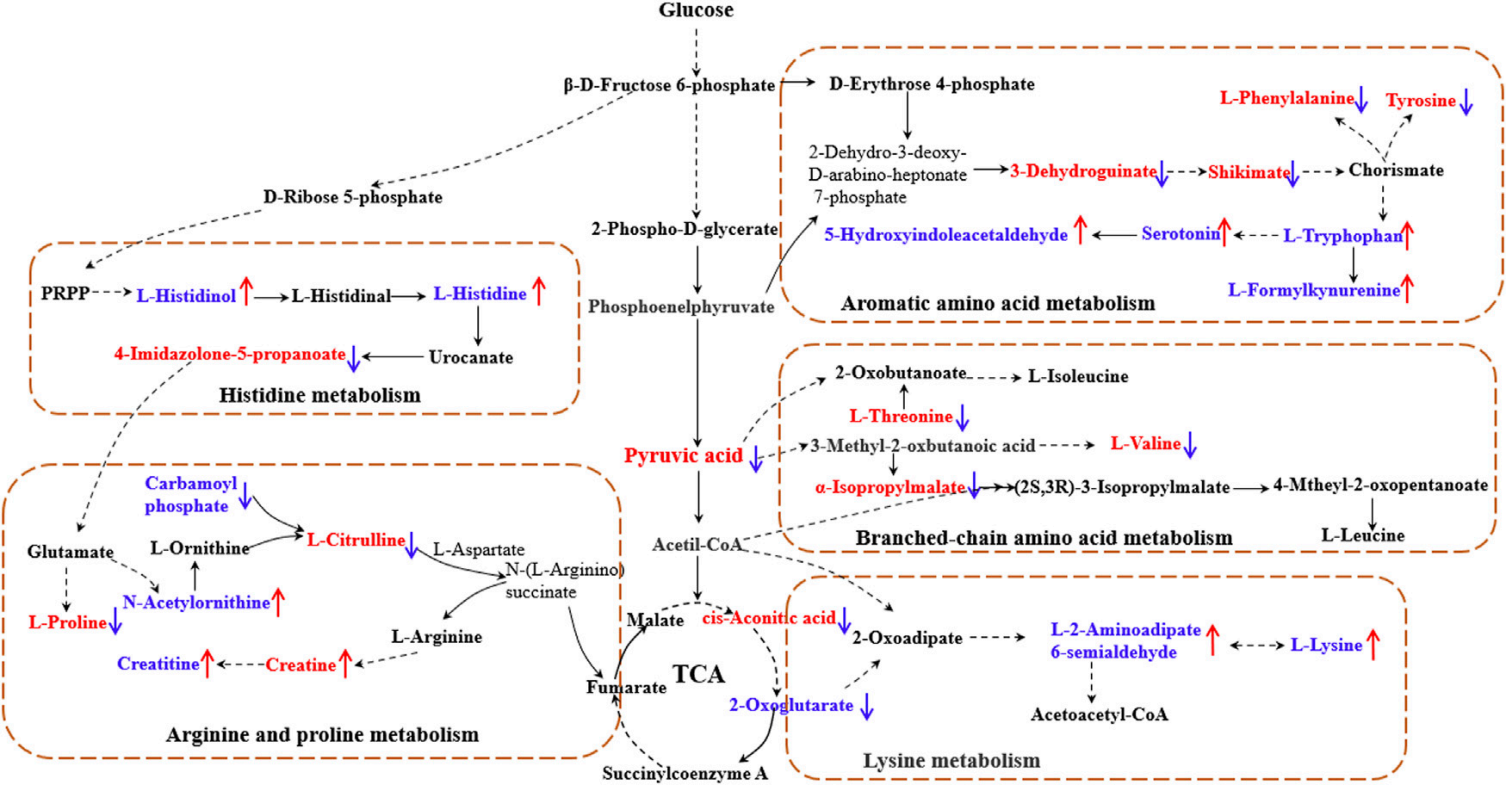
Network mechanism of MLs for the treatment of T2D (The solid line is a one-step reaction, and the dashed line is a multistep reaction. Red and blue denote differential metabolites with significantly increased and decreased contents in the serum of T2D rats."↑" and "↓" represent the differential metabolites that are increased and decreased in the serum of T2D rats after the ML treatment, respectively).

The results showed that, after treatment with MLs, the levels of citrulline, creatine, L-histidinol, pyruvic acid, cis-aconitic acid, l-phenylalanine, l-tyrosine, l-threonine, and l-valine were significantly downregulated in T2D rats, and that the l-histidine, 4-imidazolone-5-propionoate, carbamoyl phosphate, N-acetylornithine, l-proline, serotonin, 5-hydroxyindoleacetaldehyde, creatine, and l-lysine contents in the serum were upregulated (Figure 8). The metabolites were mainly involved in amino acid metabolism, glucose and lipid metabolism, and other pathways, suggesting that MLs may play a hypoglycemic role by regulating amino acid metabolism, glucose and lipid metabolism, and other pathways. A functional analysis was conducted on the amino acid metabolism pathway as well as the glucose and lipid metabolism pathways involved in the regulation of the mulberry leaf, and the network mechanism of hypoglycemia in the mulberry leaf was plotted, as shown in Figure 9. After treatment with MLs, most abnormal lipid metabolites were corrected to normal levels in T2D rats, indicating that MLs could regulate multiple lipid metabolism pathways in rats and improve IR as well as T2D symptoms.

## 4 Discussion

The incidence of T2D is increasing year by year, and it is characterized by its long duration and susceptibility to diabetic complications, which place a heavy burden on society and families (Vijan, 2019). T2D is a high-risk metabolic disease characterized by elevated blood glucose, which involves the disorder of systemic glucose metabolism, lipid metabolism, and amino acid metabolism (Roden and Shulman, 2019). Traditional Chinese medicine has rich experience in the treatment of T2D; the multicomponent and multitarget characteristics of Chinese medicine can target the complex pathogenesis of T2D and has unique advantages in the treatment of T2D. At the same time, the diverse chemical composition of Chinese medicine is a treasure trove for the exploration of hypoglycemic drugs and new drug development (Dou et al., 2021; Chu et al., 2022).

MLs are the dry old leaves of Morus alba L., and are highly effective in the treatment of type 2 diabetes (Gryn et al., 2013; Wei et al., 2018), but their specific chemical composition and hypoglycemic mechanisms are not known. In this article, the ML extracts were detected using UPLC-Q-Exactive Orbitrap-MS, and a total of 61 compounds were identified, including 16 flavonoids, 21 organic acids, nine phenolic compounds, three alkaloids, three coumarins, and nine other categories (Figure 1, Table S1). Studies have reported the pharmacological activity of several of these chemical components in regulating glucolipid metabolism and antioxidant activity, including isoquercitrin (flavonoids), protocatechuic acid, and caffeic acid (phenolic acids) with antioxidant effects (Chan et al., 2016, 2020), as well as hyperoside and rutin, which can inhibit fat formation and regulate lipid metabolism (Li J et al., 2022).

Meanwhile, we evaluated the hypoglycemic efficacy of ML extracts in T2D rats; the results showed that ML extracts can significantly reduce FBG and improve the symptoms of diabetes in T2D rats (Figure 3). The serum was rich in small-molecule metabolites, and can fully reflect the occurrence of diseases as well as the regulatory effect of drugs on the body (Tian et al., 2017). In this study, UPLC-Q-Exactive Orbitrap-MS, combined with a multivariate statistical analysis, was used to detect and analyze the changes in the metabolites in the serum. The PCA results showed that the serum metabolites of the rats in the ML group were significantly different from those of the T2D group, and tended to recover to those of the control group, suggesting that MLs were involved in regulating the metabolic process and improving the metabolic disorder in T2D rats.

Finally, 88 differential metabolites were found in the serum. As shown in Figures 8, 9, key metabolites, such as pyruvate and cisaconite, were significantly upregulated in T2D rats, suggesting that both glycolysis and the TCA cycle were abnormally upregulated in T2D rats, and that the body accelerated the absorption of dietary sugar intake. Conversely, after treatment with MLs, pyruvate and cisaconite were significantly downregulated in T2D rats. Therefore, it is suggested that MLs may reduce the intake and absorption of energy, reduce blood glucose, and improve the symptoms of T2D by inhibiting glycolysis and the TCA cycle in T2D rats.

Combined with the results of the differential metabolite pathway enrichment and the references, the results show that ML extracts increased the content of histidine in the body by regulating histidine synthesis and degradation, alleviating oxidative stress, improving chronic inflammation, and alleviating the symptoms of T2D (Visser et al., 2012; Calvani et al., 2020; Molinaro et al., 2020; Duan et al., 2021). ML extracts increase insulin sensitivity by regulating branched-chain amino acid metabolism and modulating the mTOR and AMPK signaling pathways, thereby reducing BCAA synthesis and increasing fat mobilization as well as BAT thermogenesis to improve the T2D symptoms (Luetscher, 1942; Felig et al., 1969; Felig et al., 1974). MLs may effectively treat T2D by upregulating the serum tryptophan content, improving the oxidative stress level and inflammatory response, adjusting the immune state of the body, and regulating gluconeogenesis to reduce blood glucose (Stefanović et al., 2008; Dayer et al., 2009; Ma et al., 2011; Liu et al., 2015), and MLs may achieve the purpose of treating T2D by regulating the level of lysine in the body (Bao et al., 2009; Zhang et al., 2009; Fiehn et al., 2010).

The experimental results showed that various lipid metabolites were significantly up-regulated in the serum of T2D rats, such as glycerol phosphocholine, plant sphingosine, and dihydroxyacetone phosphate. An abnormal lipid metabolism is beneficial to the development of T2D (McGarry, 2002; Han et al., 2007; Kröger et al., 2011; Floegel et al., 2013). Disorders in lipid metabolism will affect the function of pancreatic beta cells, and then cause or aggravate IR, while a deficiency in IR and insulin secretion will further aggravate lipid metabolism disorders, forming a vicious cycle, leading to the gradual aggravation of IR and insulin secretion disorders as well as lipid metabolism disorders (Engelgau et al., 2000; Wakabayashi and Daimon, 2013). After treatment with MLs, most abnormal lipid metabolites were corrected to normal levels in T2D rats, indicating that MLs could regulate multiple lipid metabolism pathways in rats and improve IR as well as T2D symptoms.

In conclusion, ML extracts contain a variety of chemical components and were able to significantly reduce fasting blood glucose in T2D rats. ML extracts may regulate multiple metabolic pathways, such as carbohydrate, amino acid, and lipid metabolism, in the body, regulate the immune state of the body, reduce oxidative stress and inflammation in the body, regulate gluconeogenesis, and increase the efficiency of fat mobilization as well as BAT thermogenesis to achieve the effect of lowering glucose and improving the symptoms of T2D.

## 5 Conclusion

In this article, the chemical composition of ML was analyzed by LC-MS and a total of 61 compounds were identified, including flavonoids, organic acids, phenolic compounds, alkaloids and coumarins, etc. The efficacy evaluation results showed that ML extracts could significantly reduce fasting blood glucose in T2D rats and improve other symptoms of diabetes. The serum metabolomics results can conclude that ML extracts improve the symptoms of T2D by regulating lipid metabolism, carbohydrate metabolism, and amino acid metabolism, regulating immune status, reducing oxidative stress and inflammation, regulating glucose metabolism, and increasing fat mobilization as well as BAT heat production.

## Data Availability

The original contributions presented in the study are included in the article/Supplementary Material, further inquiries can be directed to the corresponding authors.

## References

[B1] Ai W. He H. H. Du X. (2017). In the clinic. Type 2 diabetes. Ann. Intern. Med. 152, ITC31–ITC15. 10.7326/0003-4819-152-5-201003020-01003

[B2] Bai H. Jiang W. Wang X. Hu N. Liu L. Li X. (2021). Component changes of mulberry leaf tea processed with honey and its application to *in vitro* and *in vivo* models of diabetes. Food Addit. Contam. Part A Chem. Anal. Control Expo. Risk Assess. 38, 1840–1852. 10.1080/19440049.2021.1953709 34266375

[B3] Bao Y. Zhao T. Wang X. Qiu Y. Su M. Jia W. (2009). Metabonomic variations in the drug-treated type 2 diabetes mellitus patients and healthy volunteers. J. Proteome Res. 8, 1623–1630. 10.1021/pr800643w 19714868

[B4] Calvani R. Rodriguez-Mañas L. Picca A. Marini F. Biancolillo A. Laosa O. (2020). Identification of a circulating amino acid signature in frail older persons with type 2 diabetes mellitus: Results from the metabofrail study. Nutrients 12, 199. 10.3390/nu12010199 PMC7019630 31940925

[B5] Ceymann M. Arrigoni E. Schärer H. Baumgartner D. Nising A. B. Hurrell R. F. (2011). Rapid high performance screening method using UHPLC-MS to quantify 12 polyphenol compounds in fresh apples. Anal. Methods 3, 1774–1778. 10.1039/C1AY05152K

[B6] Chan E. W.-C. Lye P.-Y. Wong S.-K. (2016). Phytochemistry, pharmacology, and clinical trials of Morus alba. Chin. J. Nat. Med. 14, 17–30. 10.3724/SP.J.1009.2016.00017 26850343

[B7] Chan E. W. C. Wong S. K. Tangah J. Inoue T. Chan H. T. (2020). Phenolic constituents and anticancer properties of Morus alba (white mulberry) leaves. J. Integr. Med. 18, 189–195. 10.1016/j.joim.2020.02.006 32115383

[B8] Chang Y.-C. Yang M.-Y. Chen S.-C. Wang C.-J. (2016). Mulberry leaf polyphenol extract improves obesity by inducing adipocyte apoptosis and inhibiting preadipocyte differentiation and hepatic lipogenesis. J. Funct. Foods 21, 249–262. 10.1016/j.jff.2015.11.033

[B9] Chatterjee S. Khunti K. Davies M. J. (2017). Type 2 diabetes. Lancet 389, 2239–2251. 10.1016/S0140-6736(17)30058-2 28190580

[B10] Chen Z.-Z. Gerszten R. E. (2020). Metabolomics and proteomics in type 2 diabetes. Circ. Res. 126, 1613–1627. 10.1161/CIRCRESAHA.120.315898 32437301 PMC11118076

[B11] Cheng L. Wang J. An Y. Dai H. Duan Y. Shi L. (2022). Mulberry leaf activates Brown adipose tissue and induces browning of inguinal white adipose tissue in type 2 diabetic rats through regulating AMP-activated protein kinase signalling pathway. Br. J. Nutr. 127, 810–822. 10.1017/S0007114521001537 33971987

[B12] Chu N. Chan J. C. N. Chow E. (2022). Pharmacomicrobiomics in western medicine and traditional Chinese medicine in type 2 diabetes. Front. Endocrinol. 13, 857090. 10.3389/fendo.2022.857090 PMC9114736 35600606

[B13] Dayer M. R. Safari I. Dayer M. S. (2009). New evidence on hypoglycemic effect of quinolinic acid in diabetic rats. Pak. J. Biol. Sci. 12, 1025–1030. 10.3923/pjbs.2009.1025.1030 19947181

[B14] Dou Z. Xia Y. Zhang J. Li Y. Zhang Y. Zhao L. (2021). Syndrome differentiation and treatment regularity in traditional Chinese medicine for type 2 diabetes: A text mining analysis. Front. Endocrinol. 12, 728032. 10.3389/fendo.2021.728032 PMC8733618 35002950

[B15] Duan W. Zi T. Zhao Y. Shan R. Wu H. Sun H. (2021). Extent reflecting overall dietary amino acids composition adherence to the human requirement amino acids pattern is associated with the development of type 2 diabetes. Aging (Albany NY) 13, 10141–10157. 10.18632/aging.202777 33819181 PMC8064212

[B16] Engelgau M. M. Narayan K. M. Herman W. H. (2000). Screening for type 2 diabetes. United states: American Diabetes Association. 10.2337/diacare.23.10.1563 11023153

[B17] Felig P. Marliss E. Cahill G. F. (1969). Plasma amino acid levels and insulin secretion in obesity. N. Engl. J. Med. 281, 811–816. 10.1056/NEJM196910092811503 5809519

[B18] Felig P. Wahren J. Hendler R. Brundin T. (1974). Splanchnic glucose and amino acid metabolism in obesity. J. Clin. Invest. 53, 582–590. 10.1172/JCI107593 11344573 PMC301502

[B19] Fiehn O. Garvey W. T. Newman J. W. Lok K. H. Hoppel C. L. Adams S. H. (2010). Plasma metabolomic profiles reflective of glucose homeostasis in non-diabetic and type 2 diabetic obese african-American women. PLOS ONE 5, e15234. 10.1371/journal.pone.0015234 21170321 PMC3000813

[B20] Floegel A. Stefan N. Yu Z. Muhlenbruch K. Drogan D. Joost H. G. (2013). Identification of serum metabolites associated with risk of type 2 diabetes using a targeted metabolomic approach. Diabetes 62, 639–648. 10.2337/db12-0495 23043162 PMC3554384

[B21] Gryn A. Sperkowska B. Bazylak G. (2013). Determination of vitamin C and selected low molecular weight organic acids in aqueous extract of mulberry leaves used as dietary supplements. Curr. Issues Pharm. Med. Sci. 21, 226. 10.12923/J.2084-980X/26.2/A.22

[B22] Han X. Yang J. Yang K. Zhao Z. Abendschein D. R. Gross R. W. (2007). Alterations in myocardial cardiolipin content and composition occur at the very earliest stages of diabetes: A shotgun lipidomics study. Biochemistry 46, 6417–6428. 10.1021/bi7004015 17487985 PMC2139909

[B23] Hirakura K. Fujimoto Y. Fukai T. Nomura T. (2004). Two phenolic glycosides from the root bark of the cultivated mulberry tree (Morus lhou). Washington: ACS Publications. 10.1021/np50044a004

[B24] Huang Q. Tan Y. Yin P. Ye G. Gao P. Lu X. (2013). Metabolic characterization of hepatocellular carcinoma using nontargeted tissue metabolomics. Cancer Res. 73, 4992–5002. 10.1158/0008-5472.CAN-13-0308 23824744

[B26] Klejdus B. Kováčik J. (2016). Quantification of phenols in cinnamon: A special focus on “total phenols” and phenolic acids including DESI-orbitrap MS detection. Industrial Crops Prod. 83, 774–780. 10.1016/j.indcrop.2015.11.060

[B27] Kröger J. Zietemann V. Enzenbach C. Weikert C. Jansen E. H. Döring F. (2011). Erythrocyte membrane phospholipid fatty acids, desaturase activity, and dietary fatty acids in relation to risk of type 2 diabetes in the European Prospective Investigation into Cancer and Nutrition (EPIC)–Potsdam Study. Am. J. Clin. Nutr. 93, 127–142. 10.3945/ajcn.110.005447 20980488

[B28] Lee J. Ebeler S. E. Zweigenbaum J. A. Mitchell A. E. (2012). UHPLC-(ESI)QTOF MS/MS profiling of quercetin metabolites in human plasma postconsumption of applesauce enriched with apple peel and onion. J. Agric. Food Chem. 60, 8510–8520. 10.1021/jf302637t 22867437

[B29] Li J. Wu Y. Ma Y. Bai L. Li Q. Zhou X. (2022). A UPLC-MS/MS method reveals the pharmacokinetics and metabolism characteristics of kaempferol in rats under hypoxia. Drug Metab. Pharmacokinet. 43, 100440. 10.1016/j.dmpk.2021.100440 35051732

[B30] Li Q. Liao S. Pang D. Li E. Liu T. Liu F. (2022). The transported active mulberry leaf phenolics inhibited adipogenesis through PPAR-γ and Leptin signaling pathway. J. Food Biochem. 46, e14270. 10.1111/jfbc.14270 35702955

[B31] Liu J. Xiao X. Yu L. (2015). Effect of Xinjiang fermented camel milk on serum amino acid metabolism in diabetic rats. Food Industry Sci. Technol. 36, 339–342. 10.13386/j.issn1002-0306.2015.23.062

[B32] Liu Y. Li Y. Xiao Y. Peng Y. He J. Chen C. (2021). Mulberry leaf powder regulates antioxidative capacity and lipid metabolism in finishing pigs. Anim. Nutr. 7, 421–429. 10.1016/j.aninu.2020.08.005 34258430 PMC8245823

[B33] Luetscher J. A. (1942). The metabolism of amino acids in diabetes mellitus. J. Clin. Invest. 21, 275–279. 10.1172/JCI101300 16694912 PMC435140

[B34] Ma Q. Fujikura J. Ebihara K. Miyanaga F. Yokoi H. Kusakabe T. (2011). Therapeutic impact of leptin on diabetes, diabetic complications, and longevity in insulin-deficient diabetic mice. Diabetes 60, 2265–2273. 10.2337/db10-1795 21810600 PMC3161331

[B35] McGarry J. D. (2002). Banting lecture 2001: Dysregulation of fatty acid metabolism in the etiology of type 2 diabetes. Diabetes 51, 7–18. 10.2337/diabetes.51.1.7 11756317

[B36] Molinaro A. Bel Lassen P. Henricsson M. Wu H. Adriouch S. Belda E. (2020). Imidazole propionate is increased in diabetes and associated with dietary patterns and altered microbial ecology. Nat. Commun. 11, 5881. 10.1038/s41467-020-19589-w 33208748 PMC7676231

[B37] Morimoto S. Nonaka G. I. Nishioka I. (1986). Tannins and related compounds. XXXVIII, isolation and characterization of flavan-3-ol glucosides and procyanidin oligomers from Cassia bark : Cinnamomum cassia BLUME. Chem. Pharm. Bull. 34, 633–642. 10.1248/cpb.34.633

[B38] Pereira P. R. Carrageta D. F. Oliveira P. F. Rodrigues A. Alves M. G. Monteiro M. P. (2022). Metabolomics as a tool for the early diagnosis and prognosis of diabetic kidney disease. Med. Res. Rev. 42, 1518–1544. 10.1002/med.21883 35274315

[B39] Roden M. Shulman G. I. (2019). The integrative biology of type 2 diabetes. Nature 576, 51–60. 10.1038/s41586-019-1797-8 31802013

[B40] Stefanović A. Kotur-Stevuljević J. Spasić S. Bogavac-Stanojević N. Bujisić N. (2008). The influence of obesity on the oxidative stress status and the concentration of leptin in type 2 diabetes mellitus patients. Diabetes Res. Clin. Pract. 79, 156–163. 10.1016/j.diabres.2007.07.019 17850913

[B41] Thondre P. S. Lightowler H. Ahlstrom L. Gallagher A. (2021). Mulberry leaf extract improves glycaemic response and insulaemic response to sucrose in healthy subjects: results of a randomized, double blind, placebo-controlled study. Nutr. Metab. 18, 41. 10.1186/s12986-021-00571-2 PMC8047566 33858439

[B42] Tian S. Liu C. Ma S. (2017). Effect of mori folium on gene expression of TLRs in liver of diabetic mice. Chin. J. Exp. Traditional Med. Formulae 23, 145–150. 10.13422/j.cnki.syfjx.2017060137

[B43] Tian S. Wang M. Liu C. Zhao H. Zhao B. (2019). Mulberry leaf reduces inflammation and insulin resistance in type 2 diabetic mice by TLRs and insulin Signalling pathway. BMC Complement. Altern. Med. 19, 326–337. 10.1186/s12906-019-2742-y 31752797 PMC6873489

[B44] Turner B. Williams S. Taichman D. Vijan S. (2010). In the clinic. Type 2 diabetes. Ann. Intern. Med. 152, ITC31–ITC15. 10.7326/0003-4819-152-5-201003020-01003 20194231

[B45] Vieira G. S. Marques A. S. F. Machado M. T. C. Silva V. M. Hubinger M. D. (2017). Determination of anthocyanins and non-anthocyanin polyphenols by ultra performance liquid chromatography/electrospray ionization mass spectrometry (UPLC/ESI–MS) in jussara (Euterpe edulis) extracts. J. Food Sci. Technol. 54, 2135–2144. 10.1007/s13197-017-2653-1 28720971 PMC5495742

[B46] Vijan S. (2019). Type 2 diabetes. Ann. Intern. Med. 171, ITC65–ITC80. 10.7326/AITC201911050 31683294

[B47] Visser J. T. J. Bos N. A. Harthoorn L. F. Stellaard F. Beijer-Liefers S. Rozing J. (2012). Potential mechanisms explaining why hydrolyzed casein-based diets outclass single amino acid-based diets in the prevention of autoimmune diabetes in diabetes-prone BB rats. Diabetes. Metab. Res. Rev. 28, 505–513. 10.1002/dmrr.2311 22539454

[B48] Wakabayashi I. Daimon T. (2013). A strong association between lipid accumulation product and diabetes mellitus in Japanese women and men. J. Atheroscler. Thromb. 21, 282–288. 10.5551/jat.20628 24304961

[B49] Wang M. Li Y. Ma Q. (2019). Effect and mechanism of mori folium on insulin resistance in 3T3-L1 cells. Chin. J. Exp. Traditional Med. Formulae 25, 143–148. 10.13422/j.cnki.syfjx.20190128

[B50] Wang N. Zhu F. Chen L. Chen K. (2018). Proteomics, metabolomics and metagenomics for type 2 diabetes and its complications. Life Sci. 212, 194–202. 10.1016/j.lfs.2018.09.035 30243649

[B52] Want E. J. Masson P. Michopoulos F. Wilson I. D. Theodoridis G. Plumb R. S. (2013). Global metabolic profiling of animal and human tissues via UPLC-MS. Nat. Protoc. 8, 17–32. 10.1038/nprot.2012.135 23222455

[B53] Wei H. Liu S. Liao Y. Ma C. Wang D. Tong J. (2018). A systematic review of the medicinal potential of mulberry in treating diabetes mellitus. Am. J. Chin. Med. 46, 1743–1770. 10.1142/S0192415X1850088X 30518235

[B54] Wishart D. S. Feunang Y. D. Marcu A. Guo A. C. Liang K. Vázquez-Fresno R. (2018). HMDB 4.0: the human metabolome database for 2018. Nucleic Acids Res. 46, D608–D617. 10.1093/nar/gkx1089 29140435 PMC5753273

[B55] Xiong Z. Q. Li Y. Xu Y. (2021). In the clinic. Type 2 diabetes. Ann. Intern. Med. 152, ITC31–ITC15. 10.7326/0003-4819-152-5-201003020-01003

[B56] Yang J. Li Z. L. Zhou L. (2019). Multiple constituents study of Qianliexin capsule based on UHPLC-Q-Orbitrap-HRMS. Available at: https://www.acpjournals.org/doi/abs/10.7326/0003-4819-152-5-201003020-01003.

[B57] Yang Q. X. (2017). Study on chemical constituents of cinnamomi cortex. Available at: https://www.acpjournals.org/doi/abs/10.7326/0003-4819-152-5-201003020-01003 [Accessed May 20, 2022].

[B58] Yang Q. X. (2021). Study on chemical constituents of cinnamomi cortex. Guangzhou: Guangdong Pharmaceutical University.

[B59] Yi Z. H. Sun C. H. Fang H. Z. (2017). Analysis and comparison on fragmentation behavior of quercetin and morin by ESI-MS. Available at: https://www.acpjournals.org/doi/abs/10.7326/0003-4819-152-5-201003020-01003.

[B61] Zhang X. Wang Y. Hao F. Zhou X. Han X. Tang H. (2009). Human serum metabonomic analysis reveals progression axes for glucose intolerance and insulin resistance statuses. J. Proteome Res. 8, 5188–5195. 10.1021/pr900524z 19697961

