# Supplementary material for: Therapeutic mechanisms of mulberry leaves in type 2 diabetes based on metabolomics
Source: Front Pharmacol. 2022 Aug 30;13:954477. doi: 10.3389/fphar.2022.954477 (PMC9468646; doi:10.3389/fphar.2022.954477)
Supplement: Supplementary file 1 [file DataSheet1.docx]

Therapeutic Mechanisms of Mulberry Leaf in Type 2 Diabetes Rats Based on Metabolomics

**Supplementary Materials**

**Table S1** Chemical composition identification of aqueous extracts of mulberry leaves

| Number | Rt（min） | Formula | Ion mode | | ES/expected (m/z) | ES/measured (m/z) | Delta (ppm) | HPLC-ESI-MS/MS (m/z) | Identification |
| --- | --- | --- | --- | --- | --- | --- | --- | --- | --- |
| 1 | 14.60 | C_27_H_30_O_16_ | + | | 611.16066 | 611.16162 | -1.57078 | 303.05020， | Rutin |
| 2 | 15.15 | C_15_H_10_O_7_ | + | | 303.04992 | 303.05032 | -1.31991 | 229.04974, 153.01820 | Quercetin |
| 3 | 16.46 | C_15_H_10_O_6_ | + | | 287.05501 | 287.05518 | -0.59222 | 165.01877, 153.01810, 121.02863 | Kaempferol |
| 4 | 11.47 | C_15_H_10_O_6_ | + | | 287.05501 | 287.05524 | -0.80124 | 269.08179 | Luteolin |
| 5 | 16.14 | C_21_H_20_O_11_ | - | | 447.09328 | 447.09338 | -0.22367 | 284.03290, 151.00357 | Cynaroside |
| 6 | 15.89 | C_27_H_30_O_15_ | + | | 595.16574 | 595.16656 | -1.37777 | 287.05518 | Kaempferol-3-o-rutinoside |
| 7 | 14.87 | C_21_H_20_O_12_ | + | | 465.10275 | 465.10336 | -1.31154 | 303.05023 | Hyperoside |
| 8 | 14.56 | C_21_H_20_O_12_ | | - | 463.08819 | 463.08899 | -1.72753 | 301.03574 | Isoquercetin |
| 9 | 16.39 | C_21_H_20_O_11_ | | - | 447.09328 | 447.09338 | -0.22367 | 285.04038, | Quercitrin |
| 10 | 16.21 | C_21_H_20_O_11_ | | - | 447.09328 | 447.09338 | -0.22367 | 285.04062, 174.95609 | Astragalin |
| 11 | 20.13 | C_15_H_10_O_7_ | | - | 301.03537 | 301.03555 | -0.59794 | 164.92625, 135.04527 | Morin |
| 12 | 23.93 | C_19_H_18_O_5_ | | - | 325.10814 | 325.10825 | -0.33835 | 188.95163, 144.96175, 255.06648 | Moracin O |
| 13 | 15.22 | C_14_H_10_O_4_ | | - | 241.05063 | 241.05054 | 0.37337 | 199.04037, 241.05095 | Moracin M |
| 14 | 34.24 | C_25_H_26_O_6_ | | - | 421.16566 | 421.16608 | -0.99723 | 109.02946, 193.08699, 309.04065 | Kuwanon C |
| 15 | 28.97 | C_20_H_20_O_4_ | | + | 325.14343 | 325.14389 | -1.41476 | 149.02345, 167.03403, 269.08096 | Isobavachalcone |
| 16 | 11.44 | C_15_H_10_O_6_ | | + | 287.05501 | 287.05511 | -0.34837 | 201.009183, 213.09129 | Sakuranetin |
| 17 | 3.93 | C_8_H_8_O_4_ | | - | 167.03498 | 167.03462 | 2.15524 | 123.04523, 108.02171 | Vanillic acid |
| 18 | 5.10 | C_7_H_6_O_4_ | | - | 153.01933 | 153.01945 | -0.78421 | 109.0296 | Protocatechuic acid |
| 19 | 10.46 | C_9_H_8_O_4_ | | - | 179.03498 | 179.03497 | 0.05586 | 135.04529 | Caffeic acid |
| 20 | 10.33 | C_16_H_18_O_9_ | | + | 355.10235 | 355.10248 | -0.36609 | 145.02837,163.03905 | Chlorogenic acid |
| 21 | 13.00 | C_9_H_8_O_3_ | | - | 163.04006 | 163.04012 | -0.36801 | 119.05032 | 2-Hydroxycinnamic-acid |
| 22 | 19.17 | C_9_H_8_O_2_ | | + | 149.05970 | 149.06003 | -2.21388 | 131.04974, 103.05451 | trans-Cinnamic acid |
| 23 | 0.81 | C_7_H_12_O_6_ | | - | 191.05611 | 191.05603 | 0.41873 | 176.01156 | Quinic acid |
| 24 | 0.99 | C_5_H_11_NO_3_ | | - | 132.06661 | 132.06656 | 0.37860 | 115.00365 | 2,3,4-Trihydroxybenzoate |
| 25 | 2.37 | C_7_H_6_O_5_ | | - | 169.01424 | 169.01428 | -0.23667 | 125.02444 | 3,4,5-Trihydroxybenzoic acid |
| 26 | 24.00 | C_14_H_28_O_2_ | | - | 227.20165 | 227.20158 | 0.30810 | 183.1391 | Myristic acid |
| 27 | 1.00 | C_5_H_11_N_3_O_2_ | | + | 146.09240 | 146.09248 | -0.54760 | 82.06580, 100.07617, 128.07082 | 4-Guanidinobutyric acid |
| 28 | 1.18 | C_4_H_6_O_5_ | | - | 133.01424 | 133.01416 | 0.60144 | 71.0139 | Malic acid |
| 29 | 10.07 | C_7_H_10_O_5_ | | - | 173.04554 | 173.04527 | 1.56028 | 93.03457, 111.04523 | Shikimic acid |
| 30 | 0.95 | C_6_H_8_O_7_ | | - | 191.01972 | 191.01996 | -1.25641 | 111.04520, 85.02955 | Citric acid |
| 31 | 39.03 | C_18_H_30_O_2_ | | + | 279.23185 | 279.23221 | -1.28925 | 149.02347, 95.07056 | α-Linolenic acid |
| 32 | 19.82 | C_25_H_24_O_12_ | | - | 515.11949 | 515.12000 | -0.99006 | 173.05981, 135.04520 | Isochlorogenic acid C |
| 33 | 19.82 | C_25_H_24_O_12_ | | - | 515.11949 | 515.12000 | -0.99006 | 173.05981,135.04520 | Isochlorogenic acid B |
| 34 | 24.46 | C_12_H_20_O_4_ | | - | 227.12888 | 227.12889 | -0.04403 | 183.13905, 165.12839 | 9-12-Dioxododecanoicacid |
| 35 | 22.66 | C_18_H_32_O_5_ | | - | 327.21769 | 327.21783 | -0.42785 | 171.10272, 211.13412 | Corchorifatty acid F |
| 36 | 19.25 | C_15_H_20_O_4_ | | - | 263.12888 | 263.12894 | -0.22803 | 219.13919, 204.11566, 151.17648 | Abscisic acid |
| 37 | 10.03 | C_16_H_18_O_9_ | | + | 355.10235 | 355.10235 | 0.00000 | 163.03909, 193.04984 | Cryptochlorogenic acid |
| 38 | 17.63 | C_10_H_12_O_2_ | | + | 165.09100 | 165.09148 | -2.90749 | 105.07037 | Eugenol |
| 39 | 16.25 | C_22_H_26_O_8_ | | + | 419.17004 | 419.17075 | -1.69382 | 181.04973 | Syringaresinol |
| 40 | 25.33 | C_16_H_14_O_5_ | | - | 285.07684 | 285.07700 | -0.56125 | 256.95444 | Moracin A |
| 41 | 19.15 | C_19_H_18_O_5_ | | + | 327.12270 | 327.12299 | -0.88652 | 218.09366 | Moracin P |
| 42 | 31.04 | C_19_H_18_O_4_ | | - | 309.11323 | 309.11356 | -1.06757 | 291.19684, 187.09764 | Moracin C |
| 43 | 15.92 | C_14_H_12_O_4_ | | - | 243.06628 | 243.06602 | 1.06967 | 225.05576, 174.95612, 146.96126 | 4-[2-(3,5-dihydroxyphenyl) ethenyl]benzene-1,3-diol |
| 44 | 9.42 | C_26_H_32_O_14_ | | - | 567.17192 | 567.17242 | -0.88157 | 243.06654, 405.12036 | Mulberroside A |
| 45 | 9.72 | C_20_H_22_O_9_ | | + | 407.13365 | 407.13409 | -1.08073 | 227.08093, 245.08102 | Oxyresveratrol 2-O-β-D-glucopyranoside |
| 46 | 29.55 | C_17_H_26_O_4_ | | - | 293.17583 | 293.17590 | -0.23876 | 236.10539, 221.15477 | 6-Gingerol |
| 47 | 0.88 | C_6_H_13_NO_4_ | | + | 164.09173 | 164.09200 | -1.64542 | 146.08133, 69.03428 | 1-Deoxynojirimycin |
| 48 | 7.79 | C_7_H_7_NO_2_ | | - | 136.04040 | 136.04042 | -0.14702 | 136.01668 | Trigonelline |
| 49 | 15.63 | C_34_H_46_O_18_ | | - | 741.26113 | 741.26276 | -2.19896 | 161.04556, 113.02448, 71.01388 | Liriodendrin |
| 50 | 10.06 | C_9_H_6_O_4_ | | - | 177.01933 | 177.01923 | 0.56491 | 177.01942 | 5,7-Dihydroxycoumarin |
| 51 | 13.68 | C_9_H_6_O_3_ | | - | 161.02441 | 161.02444 | -0.18631 | 161.02452 | Skimmetin |
| 52 | 14.60 | C_10_H_8_O_4_ | | + | 193.04953 | 193.04984 | -1.60581 | 133.02864, 107.96033 | Scopoletin |
| 53 | 7.20 | C_9_H_10_O | | + | 135.08044 | 135.08049 | -0.37015 | 117.03379 | Cinnamyl alcohol |
| 54 | 16.42 | C_9_H_8_O | | + | 133.06479 | 133.06514 | -2.63030 | 105.07037 | Cinnamaldehyde |
| 55 | 9.66 | C_8_H_8_O_3_ | | - | 151.04006 | 151.04001 | 0.33104 | 136.01665 | Vanillin |
| 56 | 1.21 | C_6_H_14_O_6_ | | + | 181.07176 | 181.07167 | 0.49704 | 152.91771 | L(+)-Rhamnose monohydrate |
| 57 | 1.17 | C_10_H_17_NO_6_ | | + | 248.11286 | 248.11285 | 0.04030 | 230.10289 | Linamarin |
| 58 | 1.16 | C_10_H_13_N_5_O_5_ | | + | 284.09894 | 284.09946 | -1.83035 | 152.05692 | Guanosine |
| 59 | 15.89 | C_12_H_14_O_2_ | | + | 191.10665 | 191.10698 | -1.72678 | 173.13271, 117.07070 | Ligustilide |
| 60 | 28.41 | C_18_H_39_NO_3_ | | + | 318.30027 | 318.30029 | -0.06283 | 300.29025 | Phytosphingosine |
| 61 | 19.02 | C_24_H_47_NO_7_ | | + | 462.34252 | 462.34305 | -1.14634 | 300.28995, 282.27927, 264.26877 | Psychosine |

**Table S2** Fasting blood glucose（0-8 weeks）（‾*x* ± s）

| Group | Dose (g/kg) | N | Fasting blood glucose (mmol/L) | | | | |
| --- | --- | --- | --- | --- | --- | --- | --- |
|  |  |  | 0 week | 2 weeks | 4 weeks | 6 weeks | 8 weeks |
| Control | — | 10 | 5.87±0.43 | 5.8±0.8 | 5.96±1.14 | 5.15±0.47 | 5.22±0.75 |
| T2DM | — | 7 | 21.81±1.38^**^ | 26.96±4.61^**^ | 25.20±2.03^**^ | 24.40±1.53^**^ | 24.72±3.48^**^ |
| Metformin | 0.2 | 9 | 21.16±2.46 | 24.55±4.50 | 23.64±3.96 | 21.34±2.88^#^ | 17.49±3.63^##^ |
| Mulberry leaf | 4.0 | 10 | 22.45±3.22 | 26.65±3.62 | 24.69±2.41 | 21.09±3.41^#^ | 19.49±3.77^#^ |

Note: vs control, ^*^p<0.05, ^**^p<0.01, vs T2DM, ^#^p<0.05, ^##^p<0.01.

**Table S2** Fasting blood glucose（7-12 weeks）（‾*x* ± s）

| Group | Dose (g/kg) | N | Fasting blood glucose (mmol/L) | | | |
| --- | --- | --- | --- | --- | --- | --- |
|  |  |  | 9 weeks | 10 weeks | 11 weeks | 12 weeks |
| Control | — | 10 | 5.47±0.77 | 4.49±0.47 | 4.49±0.47 | 4.51±0.59 |
| T2DM | — | 7 | 23.50±2.20^**^ | 23.88±2.36^**^ | 21.84±3.27^**^ | 22.37±1.63^**^ |
| Metformin | 0.2 | 9 | 16.87±2.99^##^ | 15.20±1.28^##^ | 9.39±1.47^##^ | 9.51±2.37^##^ |
| Mulberry leaf | 4.0 | 10 | 19.43±2.77^##^ | 17.66±4.00^##^ | 14.66±3.66^##^ | 14.57±3.88^##^ |

Note: vs control, ^*^p<0.05, ^**^p<0.01, vs T2DM, ^#^p<0.05, ^##^p<0.01.

**Table S3** Fasting weight (0-6 weeks)（‾*x ±* s）

| Group | Dose (g/kg) | N | Fasting weight（g） | | | | | | |
| --- | --- | --- | --- | --- | --- | --- | --- | --- | --- |
|  |  |  | 0 week | 1 week | 2 weeks | 3 weeks | 4 weeks | 5 weeks | 6 weeks |
| Control | — | 10 | 525.47±53.27 | 540.02±55.55 | 548.68±58.07 | 560.30±57.82 | 572.64±61.81 | 599.05±60.38 | 593.43±65.43 |
| T2DM | — | 7 | 462.12±30.63^**^ | 476.63±31.57^**^ | 474.92±35.14^**^ | 472.11±44.33^**^ | 477.94±45.33^**^ | 471.02±51.42^**^ | 478.02±47.61^**^ |
| Metformin | 0.2 | 9 | 464.25±45.60 | 479.20±58.34 | 486.45±65.45 | 491.49±64.95 | 493.16±67.57 | 486.95±68.16 | 492.73±71.76 |
| Mulberry leaf | 4.0 | 10 | 460.65±75.08 | 472.62±50.86 | 475.15±53.61 | 470.26±58.05 | 474.13±56.34 | 471.10±56.76 | 473.88±53.76 |

Note: vs control, ^*^p<0.05, ^**^p<0.01, vs T2DM, ^#^p<0.05, ^##^p<0.01.

**Table S3** Fasting weight（7-12 weeks）（‾*x ±* s）

| Group | Dose (g/kg) | N | Fasting weight（g） | | | | | |
| --- | --- | --- | --- | --- | --- | --- | --- | --- |
|  |  |  | 7 weeks | 8 weeks | 9 weeks | 10 weeks | 11 weeks | 12 weeks |
| Control | — | 10 | 598.28±70.90 | 600.94±66.49 | 619.65±72.25 | 639.03±77.69 | 647.24±75.72 | 669.68±84.10 |
| T2DM | — | 7 | 485.96±51.69^**^ | 486.42±50.36^**^ | 499.93±35.52^**^ | 492.00±33.93^**^ | 458.53±26.48^**^ | 447.67±32.01^**^ |
| Metformin | 0.2 | 9 | 495.55±76.11 | 488.16±70.91 | 498.48±71.50 | 499.08±71.37 | 480.22±88.74 | 469.19±64.64 |
| Mulberry leaf | 4.0 | 10 | 475.98±52.30 | 462.40±42.85 | 466.71±45.95 | 463.85±52.13 | 443.40±45.11 | 442.74±44.94 |

Note: vs control, ^*^p<0.05, ^**^p<0.01, vs T2DM, ^#^p<0.05, ^##^p<0.01.

**Table S4** Food intake（0-6 weeks）（‾*x ±* s）

| Group | Dose (g/kg) | N | Food intake（g） | | | | | | |
| --- | --- | --- | --- | --- | --- | --- | --- | --- | --- |
|  |  |  | 0 week | 1 week | 2 weeks | 3 weeks | 4 weeks | 5 weeks | 6 weeks |
| Control | — | 10 | 27.30±1.74 | 27.35±1.63 | 28.49±3.07 | 24.71±16.74 | 27.26±1.94 | 25.87±0.33 | 27.46±1.79 |
| T2DM | — | 7 | 39.21±3.28^*^ | 43.38±3.13^*^ | 45.90±2.01^*^ | 44.16±8.81^*^ | 39.72±0.97^*^ | 35.63±8.35^*^ | 43.33±0.56^*^ |
| Metformin | 0.2 | 9 | 40.43±4.81 | 40.82±5.57 | 43.21±6.82 | 40.94±3.65 | 41.49±5.39 | 33.05±4.18 | 35.50±2.65^#^ |
| Mulberry leaf | 4.0 | 10 | 39.26±3.67 | 37.07±1.85 | 43.02±3.39 | 33.95±0.65 | 36.51±3.52 | 38.49±5.10 | 39.20±4.64 |

Note: vs control, ^*^p<0.05, ^**^p<0.01, vs T2DM, ^#^p<0.05, ^##^p<0.01.

**Table S4** Food intake（7-12 weeks）（‾*x ±* s）

| Group | Dose (g/kg) | N | Food intake（g） | | | | | |
| --- | --- | --- | --- | --- | --- | --- | --- | --- |
|  |  |  | 7 weeks | 8 weeks | 9 weeks | 10 weeks | 11 weeks | 12 weeks |
| Control | — | 10 | 24.1±1.27 | 25.86±2.29 | 28.75±0.23 | 28.43±1.8 | 27.93±1.28 | 31.15±0.93 |
| T2DM | — | 7 | 46.97±2.42^*^ | 42.22±0.41^*^ | 45.09±1.87^*^ | 46.90±1.06^*^ | 48.46±4.27^*^ | 60.43±7.34^*^ |
| Metformin | 0.2 | 9 | 39.84±4.95 | 35.05±5.94^#^ | 35.33±4.73^#^ | 39.87±3.18^#^ | 41.04±6.07^#^ | 47.08±8.45^#^ |
| Mulberry leaf | 4.0 | 10 | 33.75±2.43^#^ | 31.32±1.97^#^ | 37.74±4.38^#^ | 35.30±1.94^#^ | 42.61±3.55^#^ | 51.14±12.02 |

Note: vs control, ^*^p<0.05, ^**^p<0.01, vs T2DM, ^#^p<0.05, ^##^p<0.01.

**Table S5** water intake（0-6 weeks）（‾*x ±* s）

| Group | Dose (g/kg) | N | Water intake（mL） | | | | | | |
| --- | --- | --- | --- | --- | --- | --- | --- | --- | --- |
|  |  |  | 0 week | 1 week | 2 weeks | 3 weeks | 4 weeks | 5 weeks | 6 weeks |
| Control | — | 10 | 54.32±4.74 | 59.09±2.92 | 50.00±7.93 | 68.80±0.53 | 52.28±1.21 | 66.84±6.21 | 60.96±0.89 |
| T2DM | — | 7 | 169.54±17.28^**^ | 171.41±27.55^**^ | 185.03±15.42^**^ | 222.02±37.23^**^ | 187.54±13.2^**^ | 206.11±48.59^**^ | 206.93±9.88^**^ |
| Metformin | 0.2 | 9 | 171.06±11.30 | 152.06±10.01 | 161.39±30.86 | 160.06±21.64^#^ | 163.78±22.60 | 169.85±9.30^#^ | 192.89±15.44 |
| Mulberry leaf | 4.0 | 10 | 166.95±23.12 | 151.95±29.57 | 183.83±36.32 | 179.95±30.98 | 176.35±33.37 | 180.20±49.57 | 200.73±39.90 |

Note: vs control, ^*^p<0.05, ^**^p<0.01, vs T2DM, ^#^p<0.05, ^##^p<0.01.

**Table S5** Water intake（7-12weeks）（‾*x ±* s）

| Group | Dose (g/kg) | N | Water intake（mL） | | | | | |
| --- | --- | --- | --- | --- | --- | --- | --- | --- |
|  |  |  | 7 weeks | 8 weeks | 9 weeks | 10 weeks | 11 weeks | 12 weeks |
| Control | — | 10 | 45.38±15.20 | 71.18±13.00 | 68.41±6.64 | 53.88±4.21 | 64.76±1.18 | 46.78±2.60 |
| T2DM | — | 7 | 203.2±27.77^**^ | 210.69±25.03^**^ | 223.47±10.27^**^ | 218.61±9.18^**^ | 259.42±39.23^**^ | 305.56±37.19^**^ |
| Metformin | 0.2 | 9 | 189.96±41.10 | 193.37±18.06 | 206.00±30.39 | 164.43±11.95^#^ | 209.68±12.58^#^ | 189.67±37.50^#^ |
| Mulberry leaf | 4.0 | 10 | 194.04±33.45 | 163.74±34.64^#^ | 201.26±30.28 | 189.95±35.72 | 226.19±32.35 | 220.37±86.16^#^ |

Note: vs control, ^*^p<0.05, ^**^p<0.01, vs T2DM, ^#^p<0.05, ^##^p<0.01.

**Table S6** Differential metabolites in serum of T2D rats regulated by mulberry leaf

| NO. | Metabolites | M/Z | | RT (min) | T2DM VS Control | Mulberry leaf VS T2DM |
| --- | --- | --- | --- | --- | --- | --- |
| 1 | Pyridoxal 5'-phosphate | 285.9883 | 0.76868 | | ↑ * | ↓ # |
| 2 | Glycerophosphocholine | 280.09176 | 0.78028 | | ↑ * | ↓ # |
| 3 | Crotonic acid | 87.04454 | 23.10455 | | ↑ * | ↓ # |
| 4 | Muramic acid | 274.08932 | 0.80251 | | ↑ * | ↑ # |
| 5 | Hordatine B | 603.29846 | 3.96276 | | ↑ * | ↓ ## |
| 6 | Phytosphingosine | 318.29991 | 12.7425 | | ↑ * | ↑ ## |
| 7 | Citrulline | 176.10283 | 0.80251 | | ↑ ** | ↓ ## |
| 8 | Betaine | 118.08638 | 0.80251 | | ↑ ** | ↓ ## |
| 9 | 3-Dehydroquinate | 171.02711 | 1.50936 | | ↑ ** | ↑ # |
| 10 | Glycocholic acid | 466.31573 | 10.56058 | | ↑ ** | ↓ ## |
| 11 | Chenodeoxycholic acid | 437.29206 | 1.64838 | | ↑ ** | ↓ ## |
| 12 | Dihydroxyacetone phosphate acyl ester | 178.97966 | 1.77425 | | ↑ ** | ↑ # |
| 13 | 5-(3'-carboxy-3'-oxopropyl) -4,6-dihydroxypicolinate | 294.00278 | 0.82488 | | ↑ ** | ↓ # |
| 14 | Sulfolithocholylglycine | 512.27078 | 9.89448 | | ↑ ** | ↓ # |
| 15 | Adrenic acid | 377.2708 | 1.65986 | | ↑ ** | ↓ ## |
| 16 | Costatol | 332.92323 | 0.75163 | | ↑ ** | ↓ # |
| 17 | Tetrahomomethionine | 244.07917 | 1.86793 | | ↑ ** | ↑ # |
| 18 | L-Histidinol | 142.0972224 | 14.84763333 | | ↑ ** | ↑ # |
| 19 | Creatine | 154.0608226 | 2.914483333 | | ↑ ** | ↑ ## |
| 20 | Pyruvic acid | 87.00741159 | 2.058233333 | | ↑ ** | ↓ ## |
| 21 | cis-Aconitic acid | 155.0007172 | 31.78615 | | ↑ ** | ↓ ## |
| 22 | L-Threonine | 120.0654029 | 7.22125 | | ↑ ** | ↓ ## |
| 23 | L-Valine | 118.0860497 | 4.654616667 | | ↑ * | ↓ # |
| 24 | L-Phenylalanine | 166.08616 | 1.21808 | | ↑ * | ↓ # |
| 25 | L-Lysine | 147.11277 | 22.16275 | | ↓ * | ↑ # |
| 26 | β-Alanine | 88.03923 | 4.8015 | | ↓ * | ↑ ## |
| 27 | L-Proline | 116.07087 | 4.95928 | | ↓ * | ↑ ## |
| 28 | L-Asparagine | 131.04532 | 5.45656 | | ↓ * | ↑ ## |
| 29 | Cytosine | 112.05078 | 0.9526 | | ↓ * | ↑ # |
| 30 | N-Acetylornithine | 175.10763 | 0.85883 | | ↓ * | ↑ # |
| 31 | Pyridoxamine | 169.09465 | 0.72255 | | ↓ * | ↑ ## |
| 32 | Pyridoxine 5'-phosphate | 497.07636 | 11.01018 | | ↓ * | ↑ # |
| 33 | 4-Hydroxybenzaldehyde | 123.04417 | 1.05603 | | ↓ * | ↑ ## |
| 34 | D-Lysine | 147.11271 | 0.6995 | | ↓ * | ↑ # |
| 35 | Deoxycytidine | 455.18822 | 0.9526 | | ↓ * | ↑ # |
| 36 | L-Gulonolactone | 177.04016 | 0.9831 | | ↓ * | ↑ # |
| 37 | N-Acetyl-beta-alanine | 130.05006 | 5.10448 | | ↓ * | ↑ # |
| 38 | 2-Phenylacetamide | 136.07554 | 1.05603 | | ↓ * | ↑ ## |
| 39 | 5-Amino-6-(5'-phosphoribitylamino) uracil | 395.03873 | 0.7457 | | ↓ * | ↑ ## |
| 40 | 4-Hydroxycyclophosphamide | 551.03429 | 6.5715 | | ↓ * | ↑ ## |
| 41 | Glucoiberin | 847.06424 | 6.02036 | | ↓ * | ↑ # |
| 42 | Vicine | 285.08348 | 1.75171 | | ↓ * | ↑ ## |
| 43 | Chymostatin | 588.29699 | 14.63021 | | ↓ * | ↑ ## |
| 44 | Jadomycin B | 558.21799 | 11.0101 | | ↓ * | ↑ # |
| 45 | Cyclothiazide | 370.01184 | 6.58286 | | ↓ * | ↑ # |
| 46 | 5-Fluorouridine monophosphate | 387.02736 | 6.59428 | | ↓ * | ↑ ## |
| 47 | L-Methionine | 148.04301 | 3.01865 | | ↓ ** | ↑ # |
| 48 | L-Tryptophan | 205.0969 | 4.23118 | | ↓ ** | ↑ # |
| 49 | L-Tyrosine | 180.06635 | 1.39176 | | ↑ ** | ↓ ## |
| 50 | Taurine | 126.02198 | 2.30095 | | ↓ ** | ↓ # |
| 51 | Glyceric acid | 87.0077 | 0.9831 | | ↓ ** | ↑ # |
| 52 | L-Carnitine | 162.11232 | 0.80251 | | ↓ ** | ↑ # |
| 53 | Indolepyruvate | 248.05476 | 1.39176 | | ↓ ** | ↑ ## |
| 54 | 5-Thymidylic acid | 321.04512 | 9.27713 | | ↓ ** | ↑ ## |
| 55 | Shikimic acid | 155.03575 | 20.4843 | | ↓ ** | ↓ ## |
| 56 | Acrylic acid | 143.03565 | 20.4843 | | ↓ ** | ↓ ## |
| 57 | D-Tryptophan | 227.07887 | 4.18526 | | ↓ ** | ↑ # |
| 58 | Phosphocholine | 184.07336 | 21.31558 | | ↓ ** | ↑ ## |
| 59 | Indoleacetaldehyde | 160.07556 | 1.06696 | | ↓ ** | ↑ ## |
| 60 | Pyridine | 159.09157 | 4.8386 | | ↓ ** | ↑ # |
| 61 | Serotonin | 177.1021 | 1.06696 | | ↓ ** | ↑ ## |
| 62 | Creatinine | 114.06642 | 0.82488 | | ↓ ** | ↑ ## |
| 63 | Vasopressin | 1106.42312 | 12.04903 | | ↓ ** | ↑ ## |
| 64 | 4-Pyridoxic acid | 182.04693 | 20.4843 | | ↓ ** | ↓ ## |
| 65 | 2'-Deoxycytidine | 455.18827 | 1.7987 | | ↓ ** | ↑ ## |
| 66 | 3-Methylhistidine | 168.0773 | 9.74036 | | ↓ ** | ↑ ## |
| 67 | Galactaric acid | 248.99866 | 0.79145 | | ↓ ** | ↑ # |
| 68 | Pseudouridine | 243.06283 | 1.06406 | | ↓ ** | ↑ # |
| 69 | N'-Formylkynurenine | 259.06393 | 0.7341 | | ↓ ** | ↑ ## |
| 70 | 1-Pyrroline-4-hydroxy-2-carboxylate | 128.03432 | 2.35938 | | ↓ ** | ↑ # |
| 71 | Taurodeoxycholic acid | 498.29184 | 10.6976 | | ↓ ** | ↓ # |
| 72 | L-4-Hydroxyglutamate semialdehyde | 146.04523 | 0.80901 | | ↓ ** | ↓ # |
| 73 | Eicosapentaenoic acid | 301.21838 | 19.89748 | | ↓ ** | ↑ # |
| 74 | Kinetin | 429.15617 | 4.82983 | | ↓ ** | ↓ ## |
| 75 | L-Lathyrine | 183.08449 | 2.92615 | | ↓ ** | ↑ # |
| 76 | Pseudopurpurin | 601.05528 | 6.02036 | | ↓ ** | ↑ # |
| 77 | Phosalone | 734.97547 | 0.7457 | | ↓ ** | ↑ ## |
| 78 | 8-Bromoadenosine | 689.00111 | 0.71708 | | ↓ ** | ↑ ## |
| 79 | Tetrachlorosalicylanilide | 696.83174 | 6.77043 | | ↓ ** | ↑ ## |
| 80 | dTDP-β-L-rhodinose | 497.07766 | 0.9116 | | ↓ ** | ↑ ## |
| 81 | 9,10-DHOME | 313.23981 | 15.03213 | | ↓ ** | ↑ # |
| 82 | N-Acetyl-D-fucosamine | 206.10022 | 2.40498 | | ↓ ** | ↑ ## |
| 83 | Isoniazid alpha-ketoglutaric acid | 264.05806 | 0.94801 | | ↓ ** | ↑ ## |
| 84 | 5,6-Indolequinone-2-carboxylic acid | 192.02415 | 0.80251 | | ↓ ** | ↑ ## |
| 85 | L-Histidine | 156.0763855 | 14.15608333 | | ↓ ** | ↑ ## |
| 86 | 4-Imidazolone-5-propionoate | 157.0603219 | 2.362333333 | | ↓ ** | ↑ ## |
| 87 | Carbamoyl phosphate | 282.9767793 | 5.960083333 | | ↓ ** | ↓ ## |
| 88 | 5-Hydroxyindoleacetaldehyde | 349.1143462 | 3.466416667 | | ↓ ** | ↑ ## |

Note: "↑" and "↓" represent the increase and decrease of metabolite content respectively. Compared with control group, *p<0.05, **P<0.01, Compared with T2DM group, ^#^p<0.05, ^##^p<0.01. Mass-to-charge ratio (M/Z), retention time (RT).
